# Supplementary material for: Distorted learning from local metacognition supports transdiagnostic underconfidence
Source: Nat Commun. 2025 Feb 21;16:1854. doi: 10.1038/s41467-025-57040-0 (PMC11845503; doi:10.1038/s41467-025-57040-0)
Supplement: Supplementary file 1 — Supplementary Information [file 41467_2025_57040_MOESM1_ESM.pdf]

Distorted learning from local metacognition supports transdiagnostic underconfidence

Sucharit Katyal, Quentin JM Huys, Raymond J Dolan,

& Stephen M Fleming

Supplementary Information

Supplementary Methods

Participants

Recruitment and group allocation

In Exp 1 our goal was to have at least 25 participants per group. To achieve this, we first randomly allocated eight participants to each group to ensure the task ran successfully from beginning to end for all eight groups. We then allocated 192 participants into eight equal groups using a pre-randomised list. After exclusions, groups that lacked the minimum of 25 participants were then randomised by recruiting participants in two iterations till we reached our criterion. In all, 390 participants started Exp 1 and 314 completed it.

For our replication study, Exp 2, we determined a target sample size by calculating the number of participants that would be needed to achieve 90% power for the interaction between proportion of negative feedback trials and depression scores (PHQ-9) regressed upon intervention-block self-performance estimates. The power calculation was performed in R using the *mixedpower* package (version 0.1.0) that allows power calculations for mixed effects models[1]. Accounting for a 25% exclusion rate based on Exp 1, we estimated a sample size of 460, which we preregistered through a publicly available document (osf.io/7xfqw). When recruiting participants, we again randomised the first eight participants to ensure the task ran for all eight groups. Out of the remaining participants, we allocated group numbers for the first 448 participants using a pre-randomised list comprising eight groups equally. As some participants would start the task, receive a group allocation but subsequently drop out, any remaining participants were then allocated to one of the eight groups randomly till we reached our predetermined sample size. A total of 591 participants started Exp 2 and 460 completed it.

Exclusion

Participants were excluded from all analyses if they missed one of the three “catch” questions. Two catch questions were administered during the self-referential encoding task (SRET). Here participants were shown the words ‘human and ‘keyboard’ randomly

placed between the other 20 positive and negative adjectives; participants were excluded if they did not self-endorse these words maximally ('Yes' in Exp 1 and >.875 on the slider in Exp 2) and minimally ('No' in Exp 1 and <.125 on the slider in Exp 2) respectively. Additionally, embedded within mental health questionnaires, participants were asked "I take astronaut missions to space" and were excluded if they did not select the option "Never." In all, 14 participants were excluded in Exp 1 and 71 participants in Exp 2 for missing at least one catch question.

For analyses involving local confidence and global self-performance estimates, we also excluded participants, 1) whose performance was outside the interval (.60 .85] on any one of the 6 task blocks (Exp 1: 32 participants, Exp 2: 99 participants), 2) who did not exhibit sufficient variability in trial-by-trial confidence ratings defined as having <.05 SD across trials for each task (on a continuous confidence scale of 0—1; Exp 1: 9 participants, Exp 2: 12 participants), and 3) who did not have stable behavioural staircases in the perception/memory tasks as assessed visually (Exp 1: 19 participants, Exp 2: 0 participants). Finally, one participant from Exp 2 was excluded because their questionnaire data did not get saved on our database (possibly due to an internet issue at their end).

Note that the sample size in Exp 1 deviated slightly from our preregistered analysis. In the preregistered analysis we excluded participants whose performance was outside the closed interval (.60 .85), whereas for the reported analyses here we used a part open interval at the upper end (i.e., we included participants whose performance was exactly .85 on a block). This decision was made prior to data analysis for Exp 2 and was done because unlike Exp 1 where all blocks had 40 trials (and each correct trial would correspond to a .025 increment in accuracy), in Exp 2 the test blocks had only 20 trials (each correct trial corresponding to a .05 increase in accuracy). This would result in a more stringent exclusion criterion in Exp 2 as participants would be excluded from the study for getting only 3 (vs. 6) wrong responses on even one of the two test blocks. Consistent with this idea, we found that the preregistered criterion resulted in a performance-based exclusion of 39% of the participants compared to our expected performance-based exclusion rate of 18% from Exp 1. However, we find that even if we use the more stringent (i.e., preregistered) exclusion criterion, all our key results –

namely feedback manipulation of SPE, greater sensitivity of SPEs to low vs. high confidence with higher anxious-depression scores, lack of difference in sensitivity of SPEs to negative vs. positive feedback with higher anxious-depression scores – remain the same in both Exp 1 and 2.

In Exp 1, 19 participants were excluded as their behavioural staircases had not stabilised (no such participants were excluded in Exp 2). This was likely due to a minor error in the stimulus code, as follows. For the practice block we used a staircase with two starting "jumps" where the staircase was incremented by two steps instead of one if participants were incorrect on the first two trials. We did not intend to use these jumps for the main task blocks. However, for the first half of the participants in Exp 1 these jumps were erroneously kept as part of the main task code. This appeared to impact staircase stabilisation in a few participants who got both the initial trials incorrect who were thus excluded from analyses. For the second half of participants in Exp 1 and for all participants in Exp 2 this error was rectified.

Final sample for confidence analyses

Table S1. Number of participants in the two experiments for each of the eight groups

	Group 1	Group 2	Group 3	Group 4	Group 5	Group 6	Group 7	Group 8
Exp 1	30	26	27	30	29	27	29	32
Exp 2	36	40	32	36	32	30	32	40

The three performance-/confidence-based exclusion criteria used in the confidence analysis above were not relevant (and overly stringent) for analyses involving the self-referential encoding task (SRET). Instead, for the SRET, we used a less stringent criterion of excluding participants whose mean accuracy was below .6 across all blocks on average (and were thus presumably not paying sufficient attention to the experiment). Additionally, for this task we found that some subjects had extremely long RTs ranging from 10 seconds to several minutes. Such participants were presumably also not doing the task sincerely and would especially add noise to the pre-post feedback intervention changes in self-beliefs we aimed to test in Exp 2. We thus removed participants for whom any word RT was greater than 5 IQRs from the median

(~4.9 sec). The results were not substantially impacted by the specific criterion – similar results were obtained, for example, if we used stricter (e.g., >3 IQRs or ~3.2 sec) or more relaxed (e.g., >7 IQRs or ~6.4 sec) exclusion criteria. Final sample sizes for SRET were N = 300 for Exp 1 and N = 335 for Exp 2.

Sample-size for simulations

Our simulation procedure for model recovery was as follows. We started with N = 600 participants. For each participant we simulated an experiment for which d' values were drawn from a Gaussian distribution ($\mu = 1.1$, $\sigma = .05$) leading to a range of accuracy values. Then we excluded simulated participants whose accuracy values were outside the interval [.6 .85], to match the exclusion criterion we used on empirical data. This procedure led to ~300 participants per simulated experiment on average – a sample-size similar to Experiment 2.

Tasks

Perception and memory tasks

On the perception and memory tasks, feedback on correct and incorrect trials was accompanied by a randomly chosen message from one of three messages each.
Messages for Correct trials:

1) "Great going!"
2) "The residents of Fruitville thank you for your help!"
3) "You are getting good at this!"

Messages for Incorrect trials:

1) "You chose the wrong option!",
2) "Should have chosen the other one!",
3) "The residents of Fruitville chose the wrong fruit based on your suggestion!"

For the perception and memory tasks, we excluded trials where RTs were more than 3 IQR (inter-quartile range) away from the median RT evaluated separately for the two tasks.

Supplementary Notes

1. Interactions of *Feedback type* with *Task* and *Feedback order*

To control for potential task or order effects, we modelled 3- and 2-way interactions of *Feedback type* (positive, negative) with *Task* (perception, memory) and *Feedback order* (positive first, negative first) in predicting SPEs (see Methods), none of which were significant in Exp 1 (all p > .15). In Exp 2, there was a significant interaction between *Feedback type* and *Task* (χ^2 = 21.20, p < .0001) characterised by a larger (positive feedback > negative feedback) difference for perception compared to memory. There were no interaction or main effect of *Feedback order* in these analyses (all p > .15), allowing us to collapse over the two possible feedback orders for all subsequent analyses.

There were no interactions between *Feedback_type* and *Task* in predicting accuracy and difficulty level achieved (all p > .15).

2. Domain-general transfer of feedback to confidence

We tested if the impact of feedback on confidence transferred across distinct task domains – i.e., if there was higher confidence following positive than negative feedback blocks. In Exp 1 (Supplementary Figures 7a and 7b), we tested this effect as 3-way and 2-way interactions between *Feedback type* (on the preceding intervention block), *Task* and *Transfer type* (within domain, across domains) in predicting test-block local confidence, as follows:

$$Local\_confidence_{bc} \sim Feedback\_type * Task * Transfer\_type + Accuracy + Staircase\_level_{bc} + (1 + Feedback\_type + Accuracy + Staircase\_level_{bc} | Participant) + (1|Group)$$

We observed a trend for the 3-way interaction (χ^2 = 3.62, p = .057) characterised by a transfer effect (i.e., higher test-block local confidence following positive compared to negative feedback) within domain for both tasks, but across domain only following perception-to-memory (but not memory-to-perception) transfer (Supplementary Figure 7). The difference between test-block local confidence following positive vs. negative feedback blocks was significant for perception-to-perception transfer (t(220) = 3.24, p =

.0014), exhibited a trend for memory-to-memory transfer (t(224) = 1.61, p = .11) and a trend for perception-to-memory transfer (t(220) = 1.46, p = .15), but the opposite trend for memory-to-perception transfer (t(219) = –.73, p = .47). Visual inspection of the data indicated that transfer effects were present primarily in the first half of test blocks. We thus also performed the above analysis for the first 20 trials, this time observing a significant 3-way interaction (χ^2 = 4.8, p = .039). As before there was a significant perception-to-perception transfer ((positive – negative) estimate= .026 ± .007; t(226) = 3.57, p = .0004) and a stronger trend for memory-to-memory transfer (estimate= .014 ± .007; t(227) = 1.90, p = .059). Importantly, we observed cross-domain transfer of feedback to local confidence. Local confidence on *memory* test blocks was significantly higher following positive compared to negative feedback *perception* blocks (estimate = 0.016 ±.007; t(226) = 2.19, p = .030; Supplementary Figure 7a bottom-left). This was not the case for local confidence on perception test blocks following memory intervention blocks (estimate = –0.002 +.007; t(222) = –.28, p = .78). Generally, within-domain transfer of feedback to test-block confidence was stronger (t(227) = 3.86, p = .0001) than cross-domain transfer (t(224) = 1.38, p = .17).

Exp 2 only included measures of cross-domain transfer, with intervention blocks always being followed by test blocks of a different type. Using a similar regression model as above (without the *Transfer_type* factor), we observed a trend for an interaction between *Feedback_type* and *Task* in predicting test-block local confidence (χ^2 = 3.28, p = .070). As in Exp 1, we again found significantly greater local confidence for positive compared to negative feedback for memory test blocks following perception intervention blocks (estimate = .012 ± .004; t(275) = 3.12, p = .002; Supplementary Figures 7c and 7d) but not for perception blocks following memory blocks (estimate = 7.4e–6 ± .004; t(267) = .002, p = .999). Taken together, the two experiments demonstrate that feedback interventions impacted subsequent local confidence in a partly domain-general manner.

3. Mediation of feedback transfer to local confidence by SPE

A hierarchical model of metacognition[2] suggests that summary metacognitive beliefs are formed from local metacognitive evaluations, with more global estimates then used as priors for metacognitive evaluations across other domains or timescales. We tested if

global SPEs may act as low-dimensional summary statistics mediating the effect of feedback interventions on local confidence in subsequent test blocks. In Exp 1, a mediation analysis (Supplementary Figures 17a and 17c) confirmed that intervention-block SPE fully mediated a positive relationship between positive feedback and test-block confidence (mediated effect = 0.03, 95% CI = [.004 .06], p = .016) and negative relationship between negative feedback and test-block confidence (mediated effect = –0.06, CI = [–.12 –.01], p = .017).

Unlike in Exp 1, in Exp 2 we did not observe any significant mediation of positive and negative feedback by intervention-block SPE in predicting test confidence (both > .9), which we suspect was due to the relatively weak overall effects of confidence transfer in Exp 2 compared to Exp 1 (due to Exp 2 only including cross-domain transfer conditions). Post-hoc analyses confirmed this intuition. We found that when re-analysing Exp 1, a mediation effect was statistically reliable only when transferring to the same task (positive feedback: mediated effect = 0.04, 95% CI = [.003 .10], p = .025; negative feedback: mediated effect = –0.11, 95% CI = [–.21 –.02], p = .011) with no such mediation effect observed when transferring to the opposite task (positive feedback: mediated effect = 0.01, 95% CI = [–.02 .05], p = .38; negative feedback: mediated effect = –0.03, 95% CI = [–.09 .03], p = .35). Overall, these analyses are consistent with global confidence acting as a mediator of changes in local confidence, although this may be limited to within-domain transfer.

4. Longevity of impact of feedback on SPEs

In Exp 1, we observed that feedback effects on local confidence dissipated towards the second half of the test block (beyond ~20 trials). However, it is possible that a lower-dimensional summary of performance in the test block – an end-of-block SPE – may inherit some of the feedback effect, leading to slower-timescale dynamics in global confidence. As with local confidence on test blocks, this shift in global confidence could be domain-specific or domain-general.

For Exp 1, we first performed a factorial analysis of test-block SPEs, which showed a significant main effect of *Feedback type* (positive – negative = .030 ± .009, χ^2 = 11.71, p =

.0006), in the absence of 2- or 3-way interactions with *Task* and *Transfer type* (all $p > .2$), indicating that intervention-block feedback effects continue to exert effects on more distant test-block SPEs in a domain-general fashion (Supplementary Figure 18a). We also performed a mediation analysis to test if intervention-block SPE mediated the transfer of feedback to test-block SPE. Here again, there was a significant mediation of the effect of positive (mediated effect = 0.06, CI = [.03 .11], $p < .0001$) and negative (mediated effect = –0.15, 95% CI = [–.23 –.08], $p < .0001$) feedback on test-block SPE by intervention-block SPE (Supplementary Figures 18b and 18d).

For Exp 2, we did not find a significant main effect of *Feedback type* (positive – negative = .011 ± .009, χ^2 = 1.84, p = .18), or an interaction with *Task* (t(277) = –1.52, p = .13) on test-block SPEs, although the sign of the main effect was in the expected direction (positive > negative; Supplementary Figure 18b). Exploratory analysis however did reveal that when only considering perception-to-memory transfer, there was a significant effect of positive > negative feedback on test SPE (t(280) = 1.99, p = .048). This ordering of transfer effect magnitude (perception-to-memory > memory-to-perception) was also observed for test-block SPEs in Exp 1 (Supplementary Figure 18a). Finally, there was also no significant mediation of positive and negative feedback upon test-block SPEs by intervention-block SPEs (both $p > .8$).

These results suggest the possibility of feedback effects potentially affecting slower-timescale global confidence estimates beyond their immediate influence on local confidence, with such an effect being stronger within compared to across domains.

5. Baseline associations between mental health symptoms and confidence

In Exp 1, greater anxiety levels predicted lower mean baseline local confidence (GAD-7: χ^2 = 9.76, p = .0018; mini-SPIN: χ^2 = 8.56, p = .0034), although this was not the case for depression scores (PHQ-9: χ^2 = 1.12, p = .29; see Supplementary Figure 8 for scatter plots of confidence with mental health scores separated by *Task*). Baseline global SPEs were

also negatively predicted by both depression (PHQ-9, χ^2 = 11.87, p = .0006) and anxiety levels (GAD-7: χ^2 = 24.11, p < .0001; mini-SPIN: χ^2 = 13.74, p = .0002).

For Exp 2, we obtained transdiagnostic scores for each participant across the three transdiagnostic symptom axes, as identified by Gillan et al.[3]: anxious-depression (AD), compulsivity and intrusive thought (CIT), and social withdrawal (SW). We estimated scores along each axis using a reduced questionnaire battery developed by Hopkins et al.[4]. Replicating earlier work[5], we found that baseline local confidence was significantly negatively predicted by the A-D axis (χ^2 = 29.63, p < .0001), significantly positively by the CIT axis (χ^2 = 3.98, p = .046) and not predicted by the SW axis ($\chi^2 \sim 0$, p = .99; Figure 3d). Also consistent with recent work[6], we established that global SPEs were significantly negatively predicted by the AD axis (χ^2 = 20.26, p < .0001), but not by CIT (χ^2 = 1.42, p = .23) or SW (χ^2 = 1.21, p = .27) axes. In Exp 2, we also measured *prospective* global confidence, as self-performance estimates on the two tasks before participants performed a single trial of either task but after they were informed what the tasks would constitute. Similar to retrospective global SPEs, prospective SPEs were significantly negatively predicted by AD (χ^2 = 18.32, p < .0001), but not CIT (χ^2 = 1.07, p = .30) or SW (χ^2 = .07, p = .80) axes.

6. Choice of the "no distortion" model

Before fitting the different models of distortions in global SPEs with individual AD symptoms, we compared different accounts of group-level learning asymmetries to obtain a best fitting "no distortion" model. As in the model comparison in the main text, the learning asymmetries could be similar or different for feedback and confidence, and similar or different for the two tasks (perception/memory; see Methods). A comparison of DIC values revealed that in both Exp 1 and 2 (Supplementary Figure 19), the best fitting model was one with separate, task-specific asymmetries in learning from feedback and confidence. This model provided better fits than other model variants. This model also provided good qualitative fits to the global SPE data across 6 blocks and 8 eight groups in both Exp 1 and 2 (Supplementary Figure 20). Participants in both experiments consistently underestimated their true performance (~71% correct), a bias

which was captured by the model. Interestingly, providing biased positive feedback propelled participants' SPEs towards values that matched their performance level.

7. Local and global confidence in relation to the CIT axis

Recent work has shown that while the CIT axis is positively associated to local confidence, it is negatively related to global confidence[6]. Such a contradictory finding could be explained by individuals scoring high on the CIT axis tending to overweigh low vs. high local confidence when forming global confidence. In other words, we might expect the β_c parameter to be more negative in individuals with higher CIT scores. When fitting our model to the Exp 2 data including all three transdiagnostic axes, this is indeed what we found – that β_c (99% HDI = [–.20 –.09]) was significantly negative for the CIT axis over and above the effect observed along the AD axis, which continued to be also significantly negative (99% HDI = [–.10 –.01]; Supplementary Figure 21). Model-free analyses however did not reveal this difference as the 2-way interaction of SPE and CIT scores in predicting z-scored local confidence was not significant (χ^2 = .03, p = .87). For completeness, we also report model-fit regression slopes for the SW axis, finding that β_c was significantly greater than 0 (99% HDI = [.07 .17]). Future studies specifically targeting different symptom axes are needed to understand potential differences in global confidence formation across dimensions[6].

8. Baseline correlations of affective self-endorsements with mental health and confidence

Replicating previous work, we observed a highly significant interaction between word valence and depression and anxiety scores upon self-endorsements in Exp 1 (PHQ, χ^2 = 1310.4, p < .0001; GAD, χ^2 = 1118, p < .0001; SPIN, χ^2 = 1068, p < .0001), characterised by significant increases in self-endorsed negative words and significant decreases in self-endorsed positive words with anxious-depression symptoms (Supplementary Figure 7). In Exp 2, we found a similar interaction of word valence with the AD transdiagnostic axis (χ^2= 430.17, p < .0001; Figure 5a), which was again strongly related both to an increase in negative self-endorsements (z = 12.91, p < .0001) and decrease in positive self-endorsements (z = –15.74, p < .0001). Similar interactions were also

observed for the other two transdiagnostic axes (CIT: χ^2 = 36.78, p < .0001; SW: χ^2= 20.41, p < .0001), with the CIT interaction specifically driven by a positive relationship with negative self-endorsements (Supplementary Figures 14a–14b).

9. Preregistered SRET analysis

The analysis reported in Results slightly deviated from our preregistered analysis when assessing changes in self-beliefs with *Feedback type* (positive / negative) in Exp 2 (see Methods). As per our preregistered analysis, we also regressed *Feedback type* and its interaction with *Task* upon the double difference of word valence (positive – negative self-endorsements) and timepoint (T2 – T1). We observed a significant main effect of *Feedback type* upon the double difference of self-endorsements (t(262) = –2.05, p = .042) in the expected direction in the absence of an interaction with *Task* (t(260) =.76, p = .45). Thus, as hypothesised, the difference between positive vs. negative self-endorsements between pre- and post-feedback blocks was greater for positive compared to negative feedback.

10. Mental health scales and clinical cutoffs

In Exp 1, we used standardised clinical scales for depression (PHQ-9) and general anxiety (GAD-7). Supplementary Figure 22 shows histograms of scores on the PHQ and GAD separate by clinical cutoffs for these scores. Median scores for both PHQ and GAD were 4 (black vertical line in Supplementary Figure 22) indicating that nearly half the participants had greater than minimal severity for depression and anxiety. According to Kroenke and Spitzer[7], for the PHQ-9, the single recommended cut-off point is ≥10 with a sensitivity of 88% for major depression with a specificity of 88%. In Exp 1, 24.3% of the participants had PHQ scores ≥10. Similarly, for the GAD-7, the single recommended cutoff is again ≥10 (sensitivity 89%, specificity, 82%); 20% of Exp 1 participants had GAD scores ≥10.

In Exp 2, we used a questionnaire battery that included subsets of questions from different standard clinical questionnaires to extract transdiagnostic symptom

dimensions[4]. Because the full depression and anxiety questionnaires were not collected, we could not evaluate our sample in terms of standard clinical cutoffs.

11. Participants' awareness of study manipulations

11.1. Awareness of feedback manipulation

At the end of the study, we asked participants four debriefing questions about the study. Two questions were about if they noticed that the "auditor" was biased on certain blocks to give feedback more often on correct than incorrect and incorrect than correct trials. In both experiments (Supplementary Figure 23a and 23d), more participants reported noticing biased feedback on negative feedback blocks (Exp 1: 67.4% of participants, Exp 2 70.2%) than positive feedback blocks (Exp 1: 54.0% of participants, Exp 2 57.8%). This is expected because participants performed above chance and would thus not expect more negative feedback. There was however no difference in AD scores between participants who reported being aware vs. not aware of biased positive feedback blocks (Exp 1: $t(208) = -.39$, $p = .70$; Exp 2: $t(249) = .84$, $p = .40$) or between participants who reported being aware vs. not aware of biased negative feedback blocks (Exp 1: $t(148) = .21$, $p = .84$; Exp 2: $t(158) = -1.85$, $p = .07$).

11.2. Feedback-induced mood shifts

We also asked participants how they felt upon receiving correct and incorrect feedback (Supplementary Figure 23b and 23e). In both experiments, a majority of participants reported feeling better after receiving positive feedback (Exp 1: 70.4% of participants, Exp 2 65.8%) and worse after receiving negative feedback (Exp 1: 66.1% of participants, Exp 2 71.2%). In both experiments, participants who felt worse after negative feedback had higher anxious-depression scores than participants who did not feel worse (Exp 1: GAD-7 – W(Wilcoxon rank sum test) = 7088, $p = .015$; PHQ-9 – W = 6697, $p = .11$; Exp 2: AD axis – W = 9968, $p = .0007$; Supplementary Figure 23c and 23f). However, these scores did not differ between participants who felt better after positive feedback compared to those who did not feel better (Exp 1: GAD-7 – W = 6208, $p = .13$; PHQ-9 – W = 5705, $p = .67$; Exp 2: AD axis – W = 9182, $p = .44$).

12. Metacognitive efficiency not related to mental health symptoms

We explored if metacognitive efficiency, as measured by M-ratio (meta-d'/d')[8], was related to mental health scores. We calculated M-ratio using the HMeta-d toolbox[9] for the baseline blocks separately for each subject and task. We then used the following regression model to assess if mental health scores (MHS) predicted M-ratio:

$$M\_ratio \sim MHS + Age + Gender + (1|Task)$$

In Exp 1, neither PHQ (χ^2 = .07, p = .79) nor GAD (χ^2 = .63, p = .43) scores predicted M-ratio. Similarly, in Exp 2, none of AD (χ^2 = 2.60, p = .11), CIT (χ^2 = 3.18, p = .08) or SW (χ^2 = 2.34, p = .13) axes predicted M-ratio. These results are consistent with previous literature, which shows a lack of relationship between metacognitive efficiency measured using M-ratio and mental health symptoms[5,10].

13. No moderation of feedback's effect on SPE by metacognitive efficiency

We also explored if individuals' metacognitive efficiency moderated the impact of feedback on global SPEs. To do so we tested if 3-way interaction of M-ratio with *Feedback_type* and *Task*, and 2-way interaction of M-ratio with F*eedback_type* predicted baseline-corrected SPEs for the non-baseline blocks using the following model:

$$SPE_{bc} \sim Feedback\_type * Task * M\_ratio + Accuracy_{bc} + Staircase\_level_{bc} + (1|Participant)$$

In both Exp 1 and 2, we did not observe any 3- or 2-way interactions (all p > .38) indicating that M-ratio did not moderate the impact of feedback on global SPEs.

Supplementary Figures

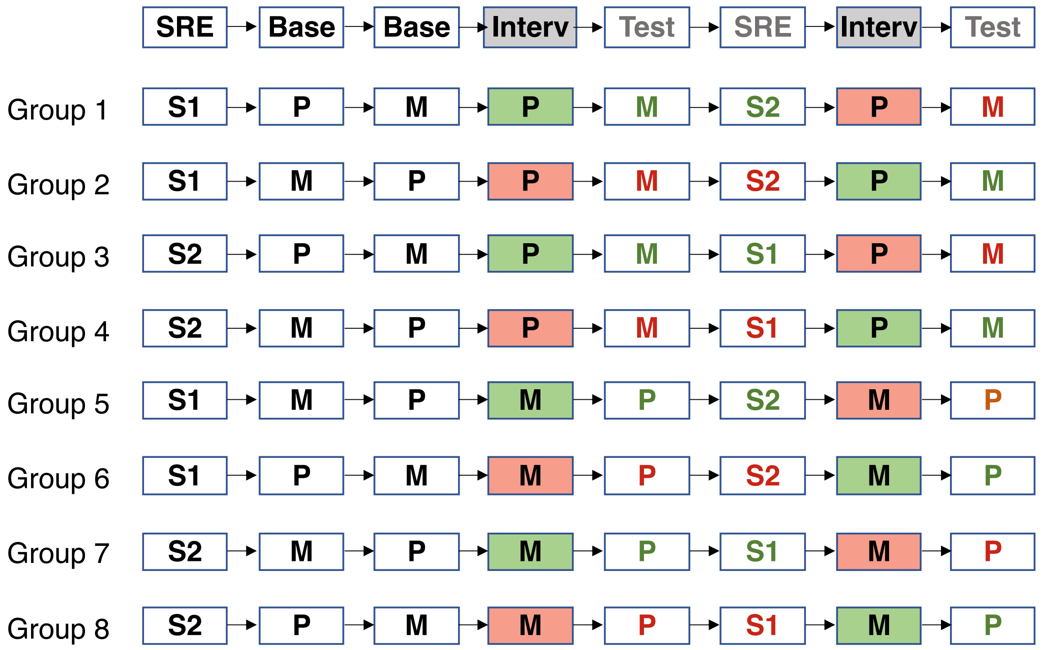

Supplementary Figure 1. Eight possible groups of participants in Exp 2 with the order in which they performed the three tasks, perception (P), memory (M) and self-referential encoding (SRE; with one of two sets of words, S1 or S2) task. The study started with one block of the SRE task. This was be followed by two baseline blocks of the perception and memory task (order randomised across participants). Next, on the intervention block (Interv) they were provided either more frequent positive (green) or negative (red) feedback. They then underwent a test block of the opposite task from the intervention block. Next, they were administered the SRE task with the other set of words from the baseline SRE block. Finally, they performed another set of intervention and test blocks where feedback on the intervention block was opposite to the feedback (more negative or more positive) given on the first intervention block.

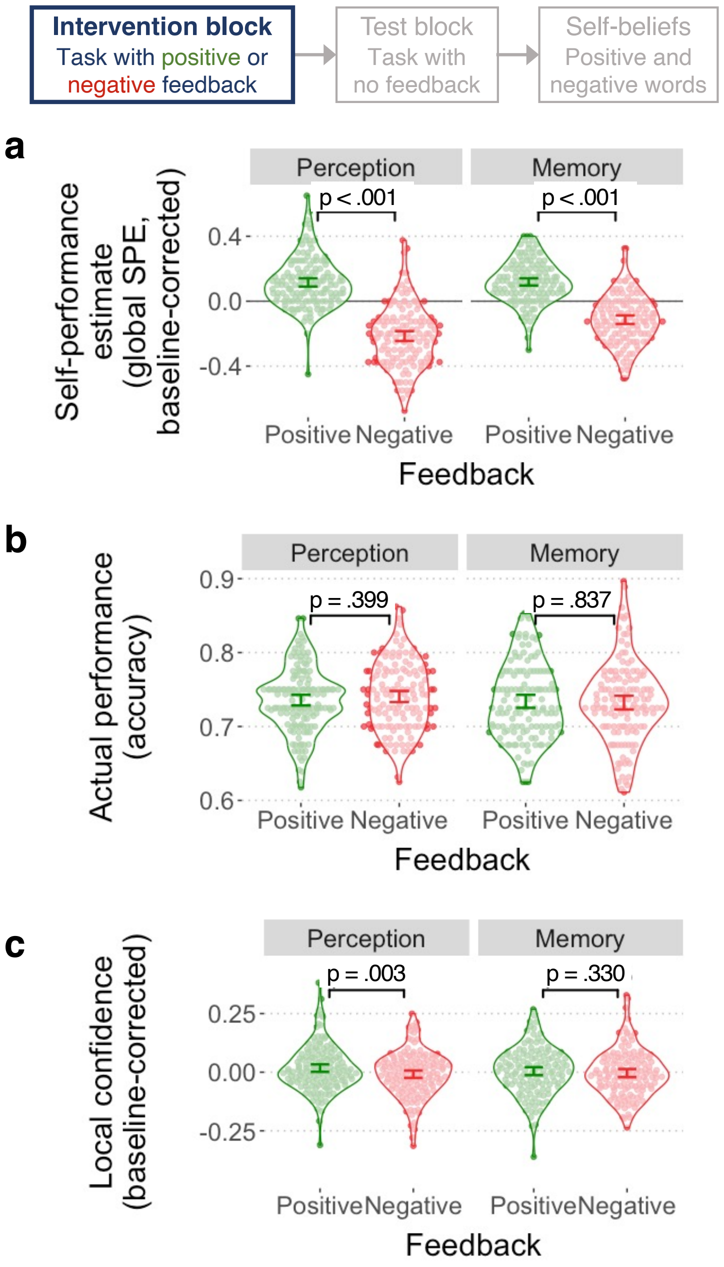

Supplementary Figure 2. The effect of positive (green) and negative (red) feedback on **a)** global SPEs (baseline subtracted), **b)** accuracy, and **c)** local confidence (baseline subtracted) of the intervention block for the two tasks in Exp 2. Each dot is individual participant data (N = 278). Error bars show 95% bootstrapped confidence intervals, statistical comparisons were performed using mixed regressions, and p-values were obtained through likelihood ratio tests with χ^2 distributions.

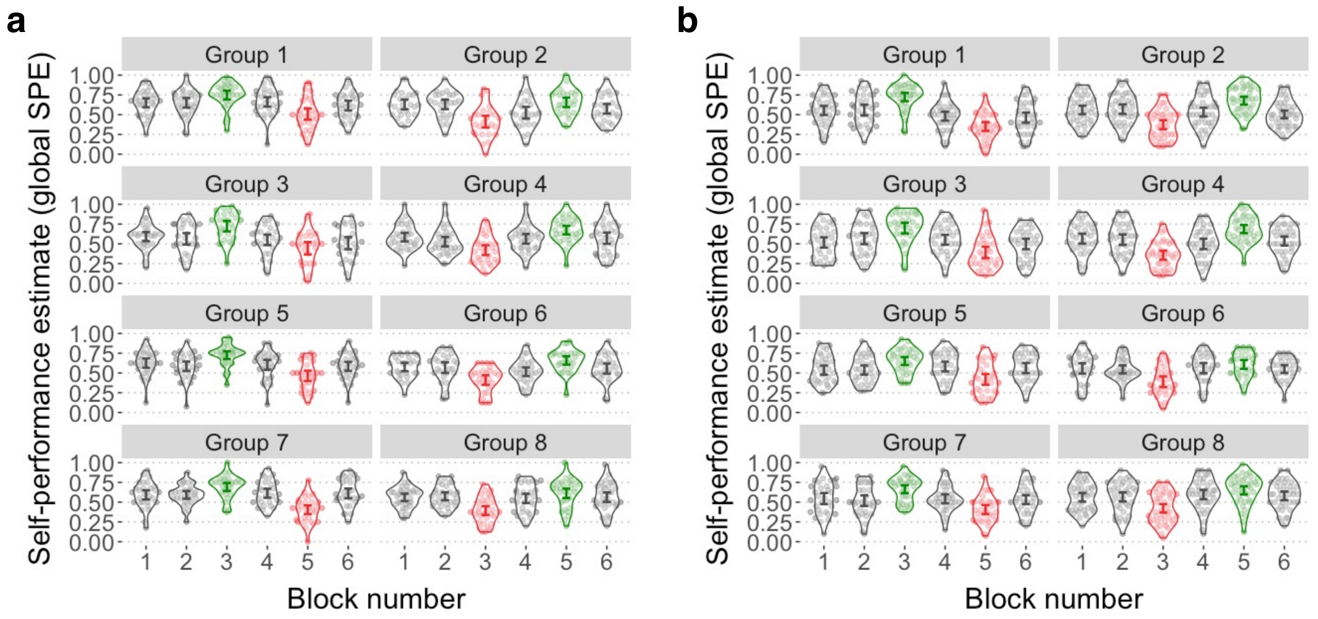

Supplementary Figure 3. Global SPEs for the eight groups of participants with positive feedback blocks in **a)** Exp 1 (N = 230), **b)** Exp 2 (N = 278). Blocks are colour-coded for feedback (grey – no feedback, green – positive feedback, red – negative feedback). Each dot in the cloud is a single participant and error bars indicate 95% bootstrapped confidence intervals.

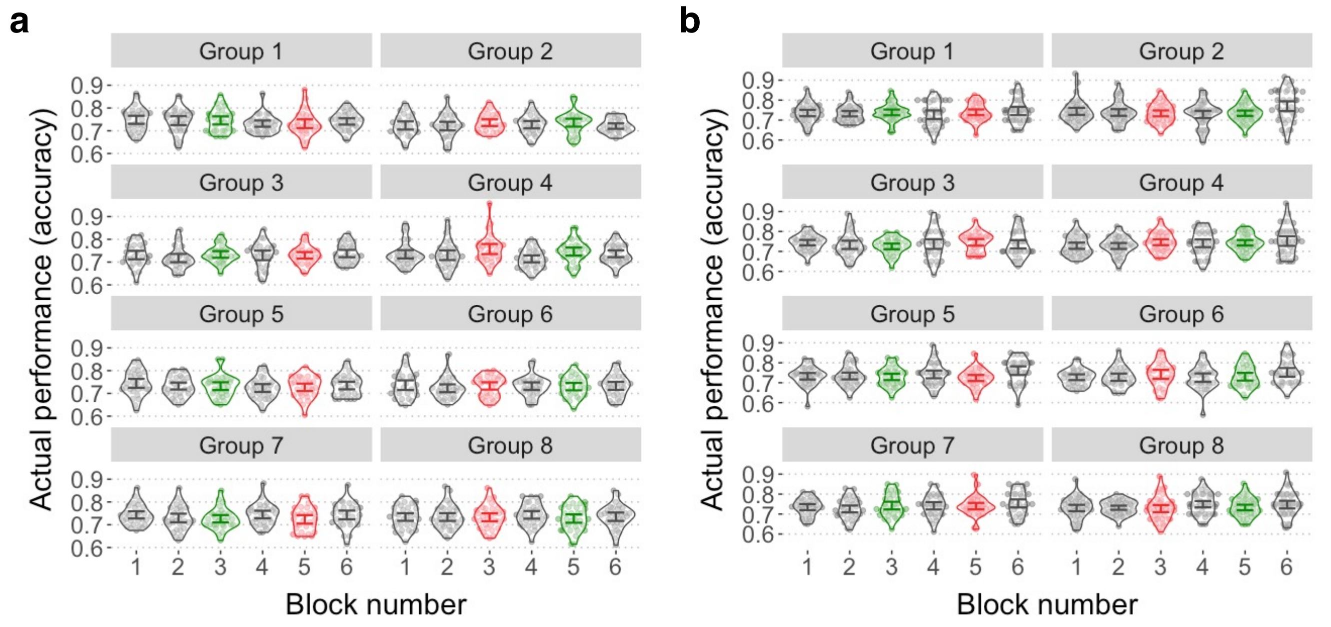

Supplementary Figure 4. Performance (task accuracy) for the eight groups of participants with positive feedback blocks in **a)** Exp 1 (N = 230), **b)** Exp 2 (N = 278). Blocks are colour-coded for feedback (grey – no feedback, green – positive feedback, red – negative feedback). Each dot in the cloud is a single participant and error bars indicate 95% bootstrapped confidence intervals.

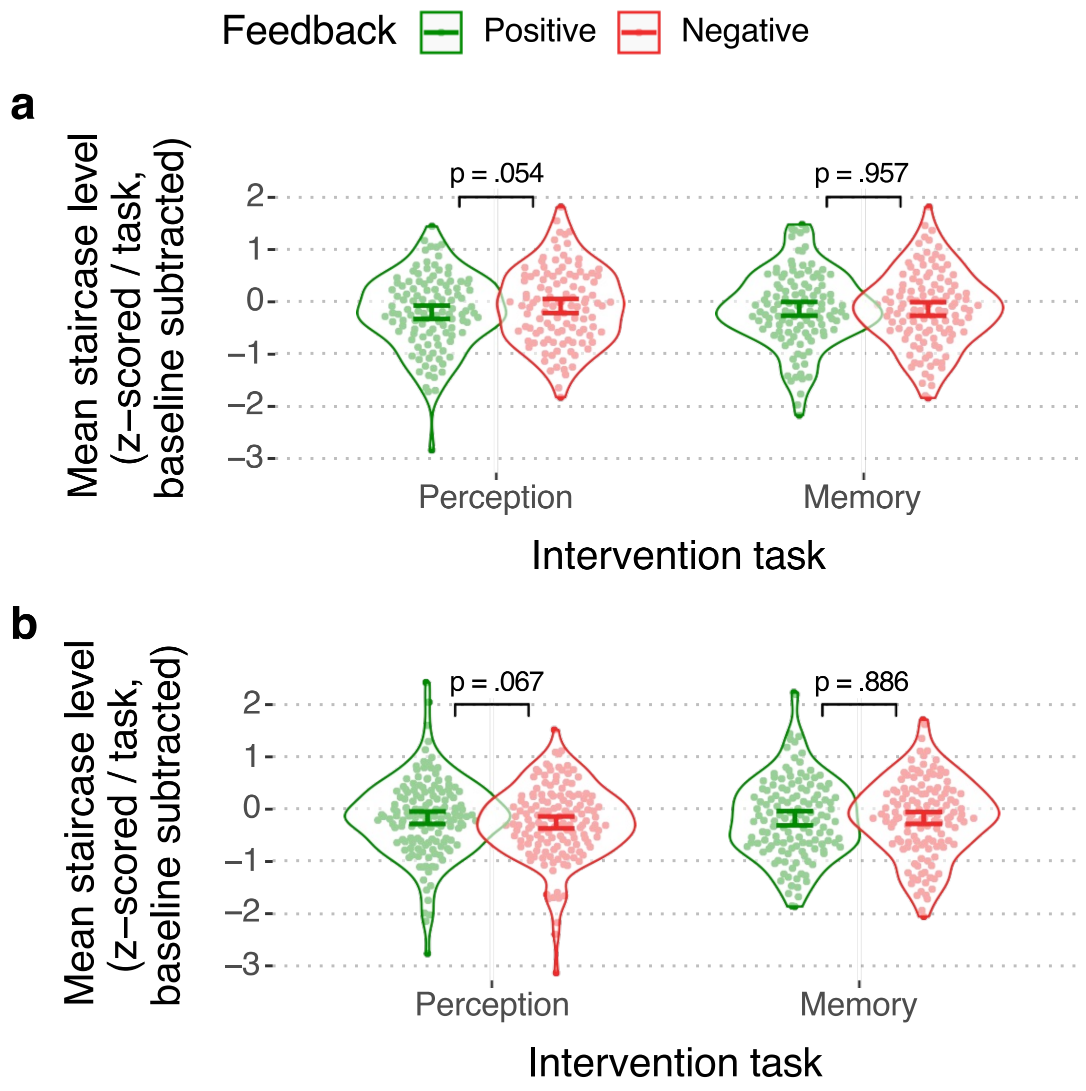

Supplementary Figure 5. The effect of positive (green) and negative (red) feedback on average difficulty achieved (mean staircase level) of the intervention block for the two tasks in **a)** Exp 1 (N = 230) and, **b)** Exp 2 (N = 278) plotted separately for the two tasks Each dot is individual participant data. Error bars show 95% bootstrapped confidence intervals, statistical comparisons were performed using mixed regressions, and p-values were obtained through likelihood ratio tests with χ^2 distributions.

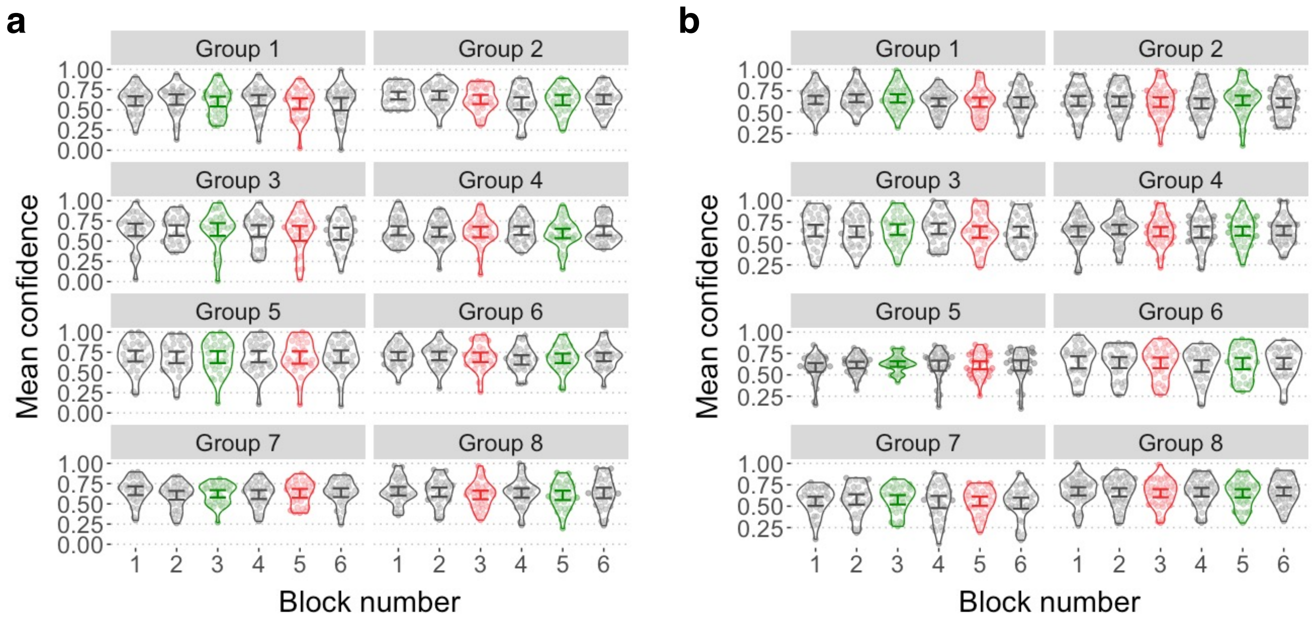

Supplementary Figure 6. Mean local confidence for the eight groups of participants with positive feedback blocks in **a)** Exp 1 (N = 230), **b)** Exp 2 (N = 278). Blocks are colour-coded for feedback (grey – no feedback, green – positive feedback, red – negative feedback). Each dot in the cloud is a single participant and error bars indicate 95% bootstrapped confidence intervals.

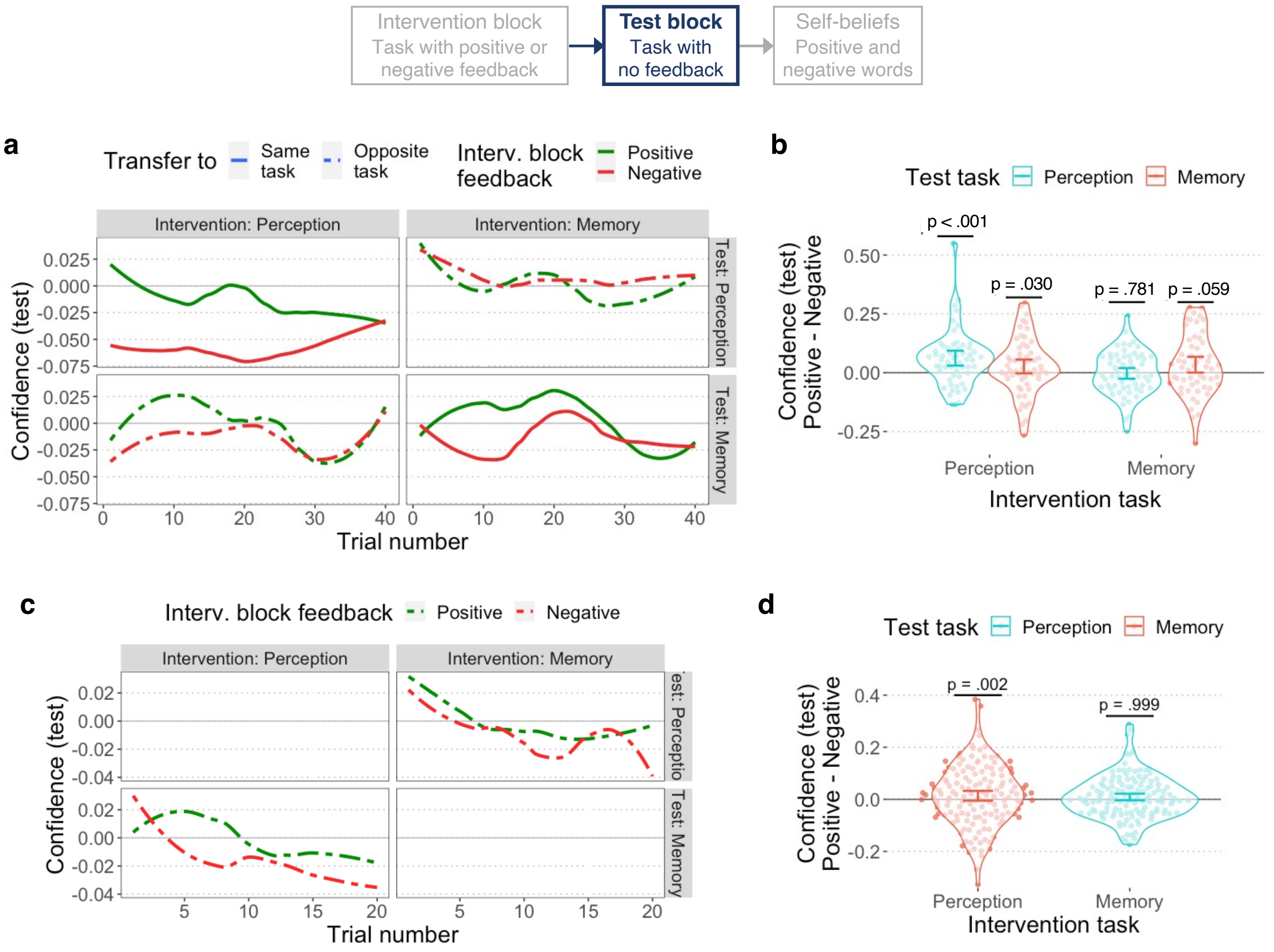

Supplementary Figure 7. Transfer of feedback during intervention blocks to local confidence during test blocks for **a, b)** Exp 1 (N = 230) and **c, d)** Exp 2 (N = 278). **a, c)** Mean local confidence on test blocks plotted as a function of trial number (smoothed using a local regression (loess) span of 0.6) for positive (green) and negative (red) feedback blocks plotted separately for the two intervention tasks in panels along the columns, and test tasks along the rows. **b, d)** Difference in mean confidence of test blocks following positive and negative intervention blocks for the two intervention tasks (x-axis) and two test tasks (colours). Note that in Exp 2 there was no transfer to the same task and only to the opposite task. For panels (b) and (d), error bars show 95% bootstrapped confidence intervals, statistical comparisons were performed using mixed regressions, and p-values were obtained through likelihood ratio tests with χ^2 distributions.

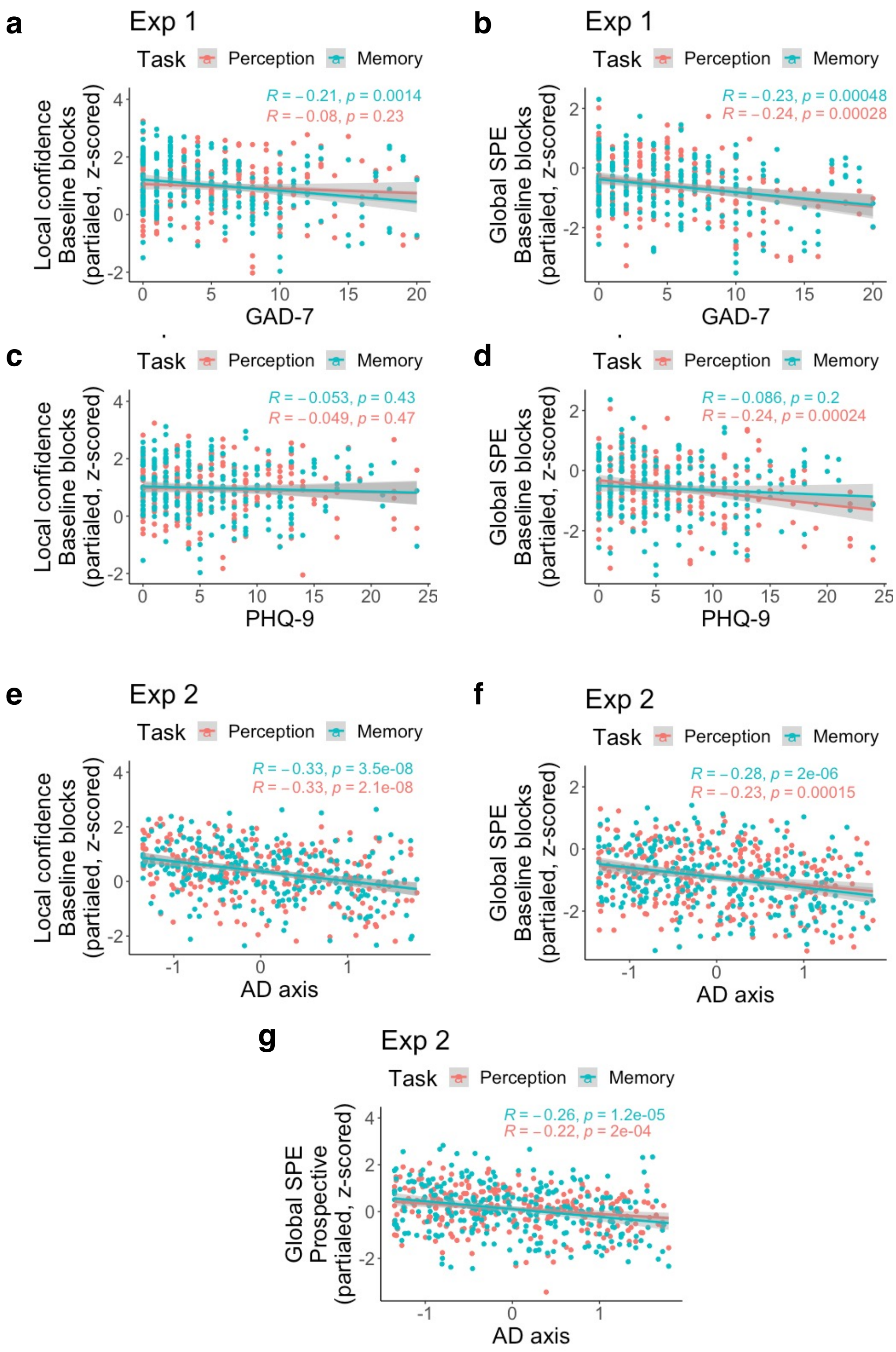

Supplementary Figure 8. Scatter plots depicting baseline relationships between anxious-depression scores and confidence in **a—d)** Exp 1 (N = 230), and **e—g)** Exp 2 (N = 278) separated by task. **a, c, e)** Local confidence. **b, d, f)** Global SPEs. **g)** Prospective global SPEs. Note that y-axes depict partialled confidence values from regression models from which effects of age, gender, accuracy, difficulty and response times have been regressed out. Shaded error bars show 95% bootstrapped confidence intervals.

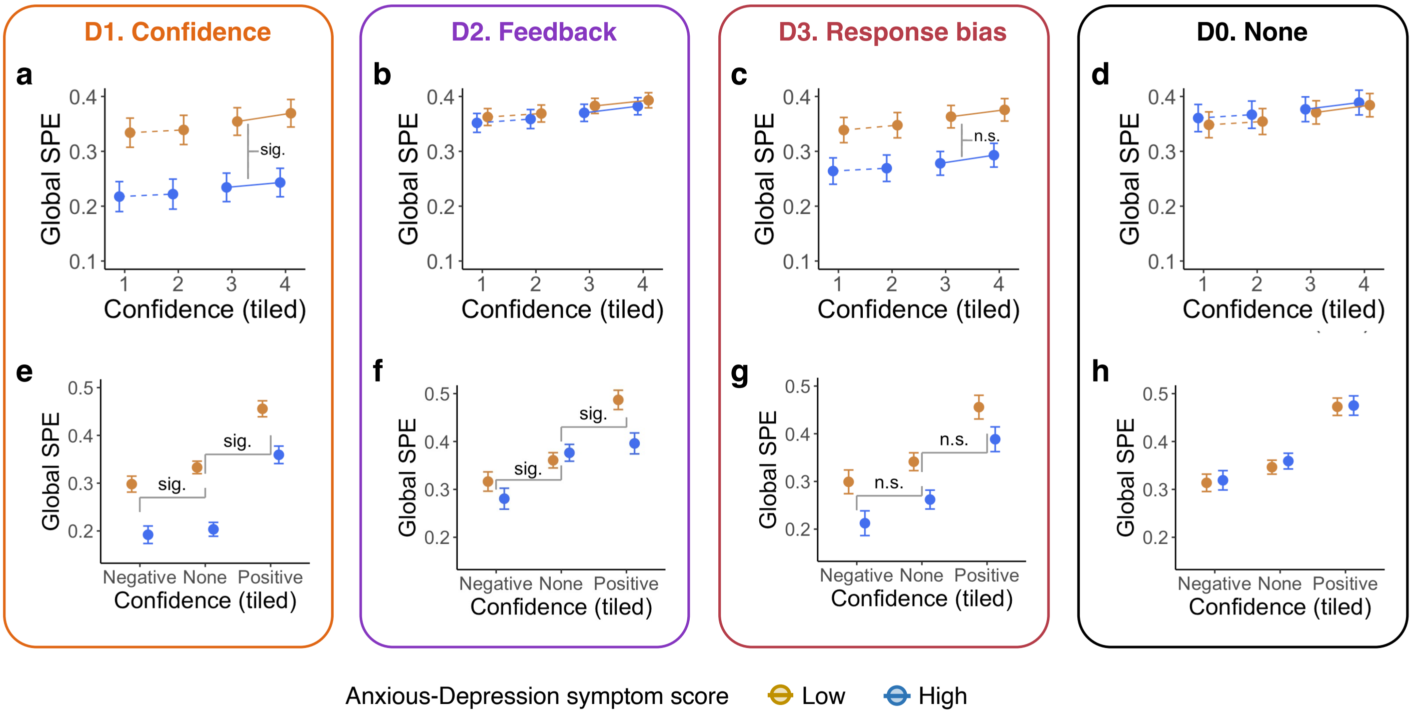

Supplementary Figure 9. Simulated data from different models, organised akin to main text Figures 1e–1f and Figures 4a–4d, depicting our qualitative statistical predictions. **a, e)** Confidence distortion model where increasing anxious-depression symptoms are associated with a blunted effect of higher local confidence on global SPEs. **b, f)** Feedback distortion model where higher anxious-depression symptoms are associated with global SPEs being more sensitive to negative feedback, and lower symptoms more sensitive to positive feedback. **c, g)** Response bias model where higher anxious-depression symptoms are associated with an overall decrease in global SPEs (but no differences in learning). **d, h)** The null model where global SPEs do not depend on anxious-depression symptoms. Note that the small but significant interaction effects between symptoms and feedback type predicted under the confidence distortion model (e) are in the opposite direction to those predicted under the feedback distortion model (f).

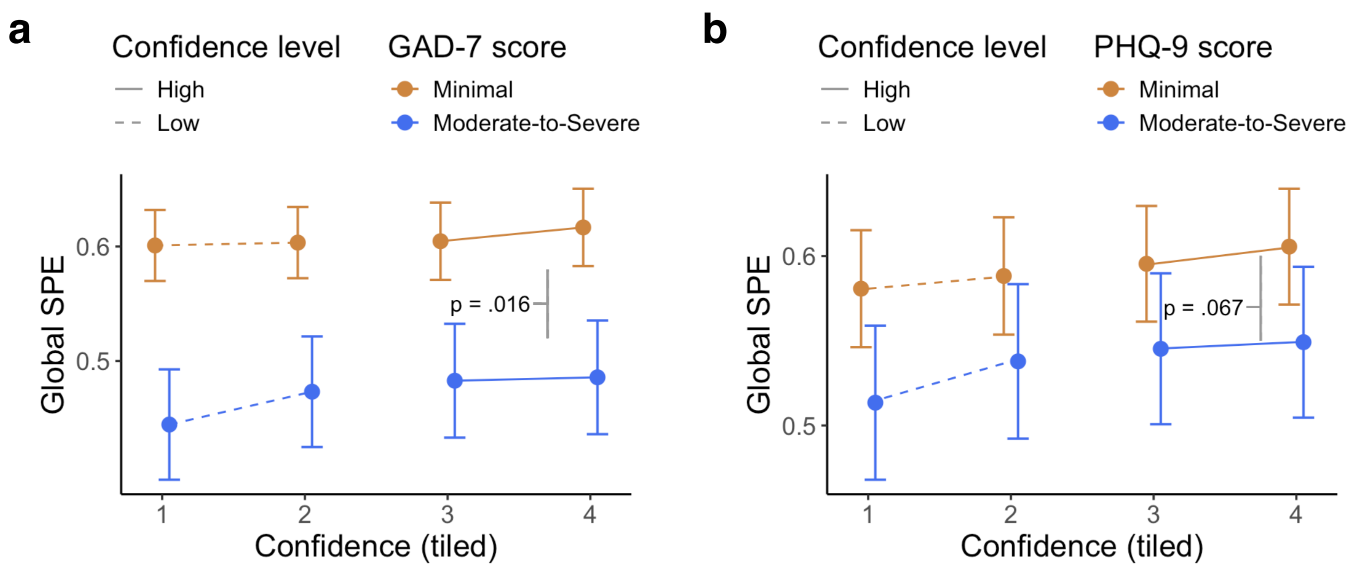

Supplementary Figure 10. Data from Exp 1 plotted similar to main text Figure 4A except that instead of depicting the means of the highest and lowest tertiles of anxious depression symptoms, we separate data into two groups—minimal risk (gold) and moderate-to-severe risk (blue)—based on clinical cutoffs for the **a)** GAD-7, and **b)** PHQ-9 scales (see Supplementary Figure 22 for symptom score distributions based on clinical cutoffs). For statistical analysis, we used this grouping factor based on symptom scores (instead of continuous anxiety and depression scores in our main analyses) and regressed its interaction with global SPEs upon local confidence. We report in these plots p values for these 2-way interactions, which were in the expected direction for both GAD-7 and PHQ-9, though significant at alpha = .05 only for GAD. Error bars show 95% bootstrapped confidence intervals, statistical comparisons were performed using mixed regressions, and p-values were obtained through likelihood ratio tests with χ^2 distributions.

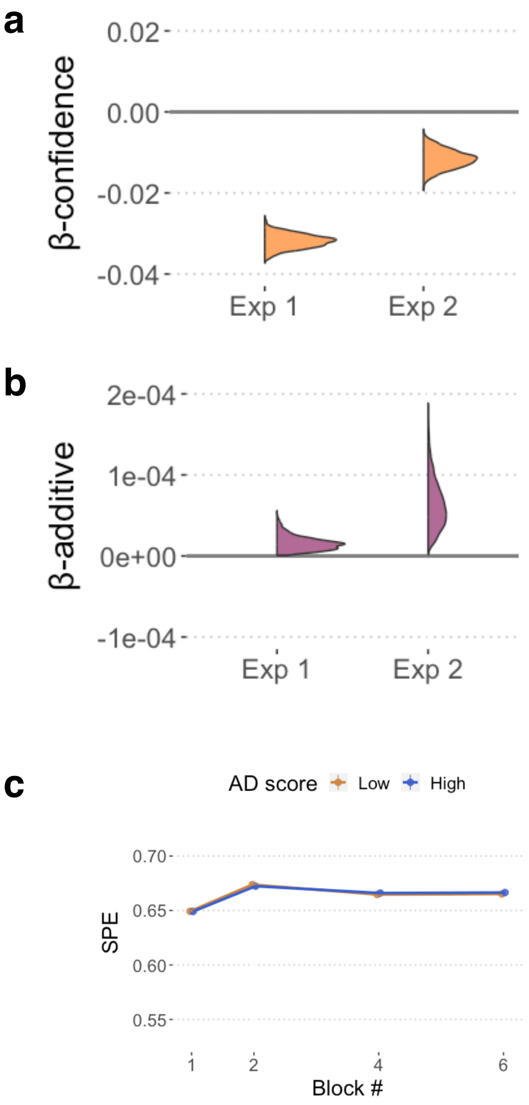

Supplementary Figure 11. Posterior distributions of regression slope parameters for model D5 relating anxious-depression symptoms simultaneously to **a)** confidence distortion and **b)** response bias in Exp 1 and Exp 2. Regression coefficient values from Exp 2 are divided by 5 (corresponding to differences in the range of PHQ and AD axis scores) to allow direct comparison of coefficients. **c)** A simulation of the difference in global SPEs with low and high AD scores where the response bias is of the order found in Exp 1 (similar results for Exp 2) showing little impact of anxious-depression scores on global SPEs with just response bias.

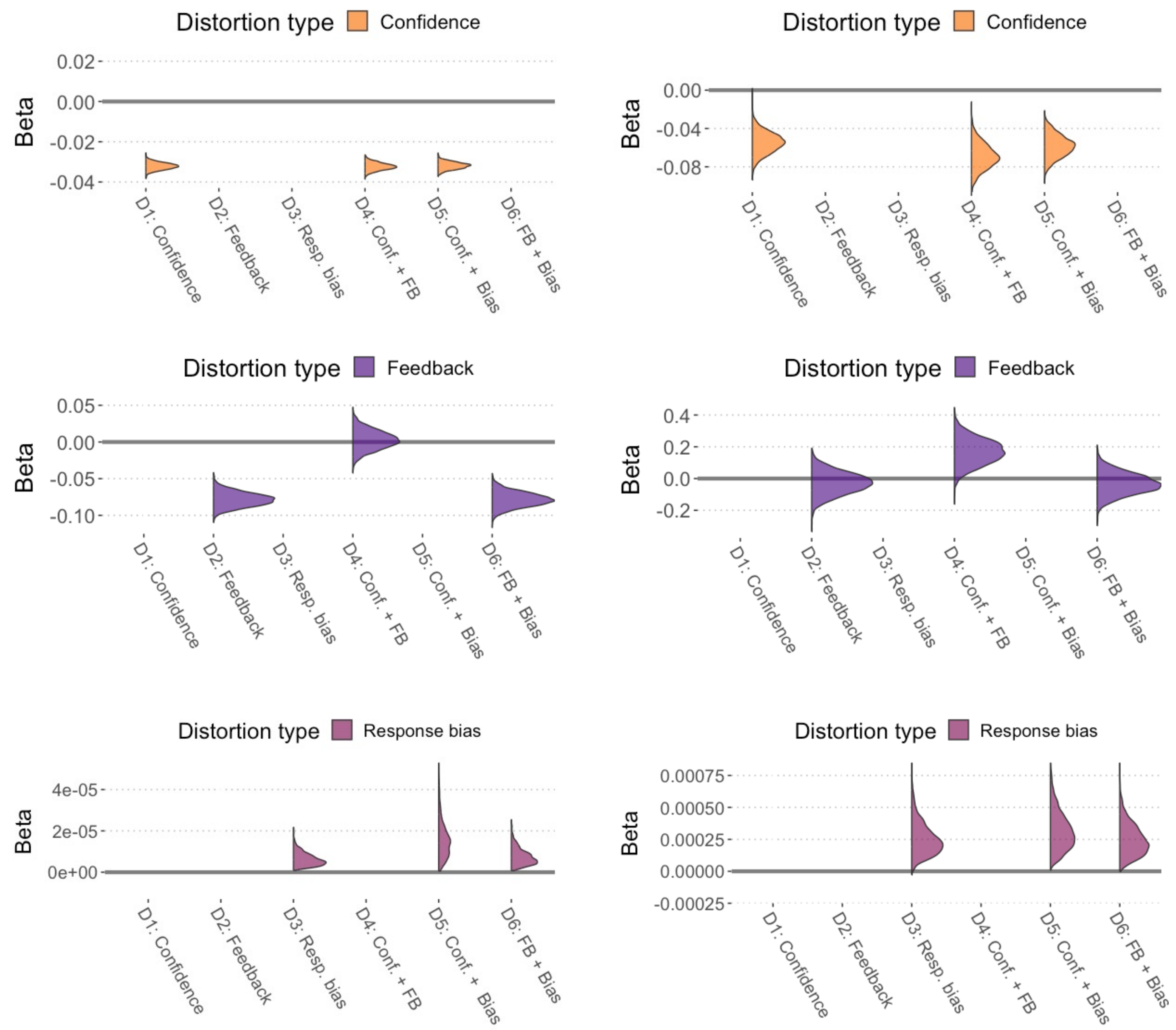

Supplementary Figure 12. Symptom regression betas estimates from Exp 1 (left) and Exp 2 (right) for confidence (top), feedback (middle) and response bias (bottom) distortions for all 6 tested models (i.e., where distortions were modeled individually and together two at a time).

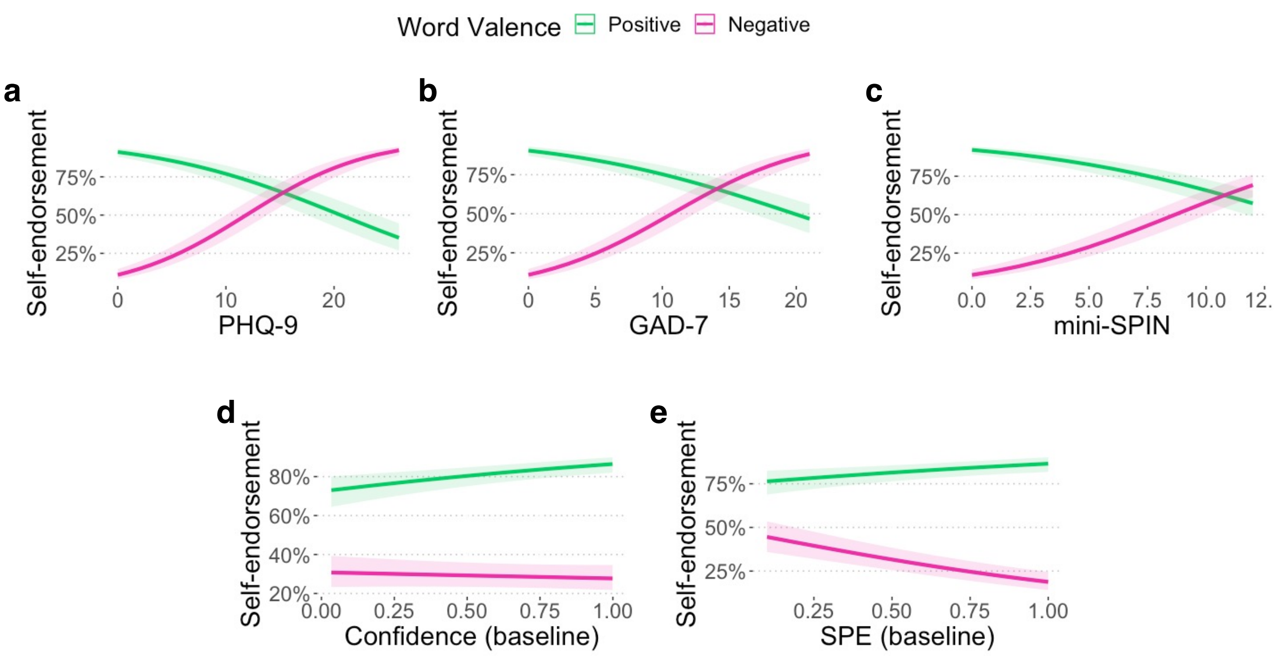

Supplementary Figure 13. Valence-specific relationship of self-endorsement of positive and negative word during the self-referential encoding task in Exp 1 (N = 300) plotted as marginal effects from a logistic regression showing that **a)** PHQ-9, **b)** GAD-7, and **c)** mini-SPIN scores are negatively related to positive word endorsement and positively related negative word endorsement. Similar plots in Exp 1 for **d)** global SPEs, and **e)** mean local confidence during baseline showing that they are associated positively with positive and negatively with negative word endorsements.

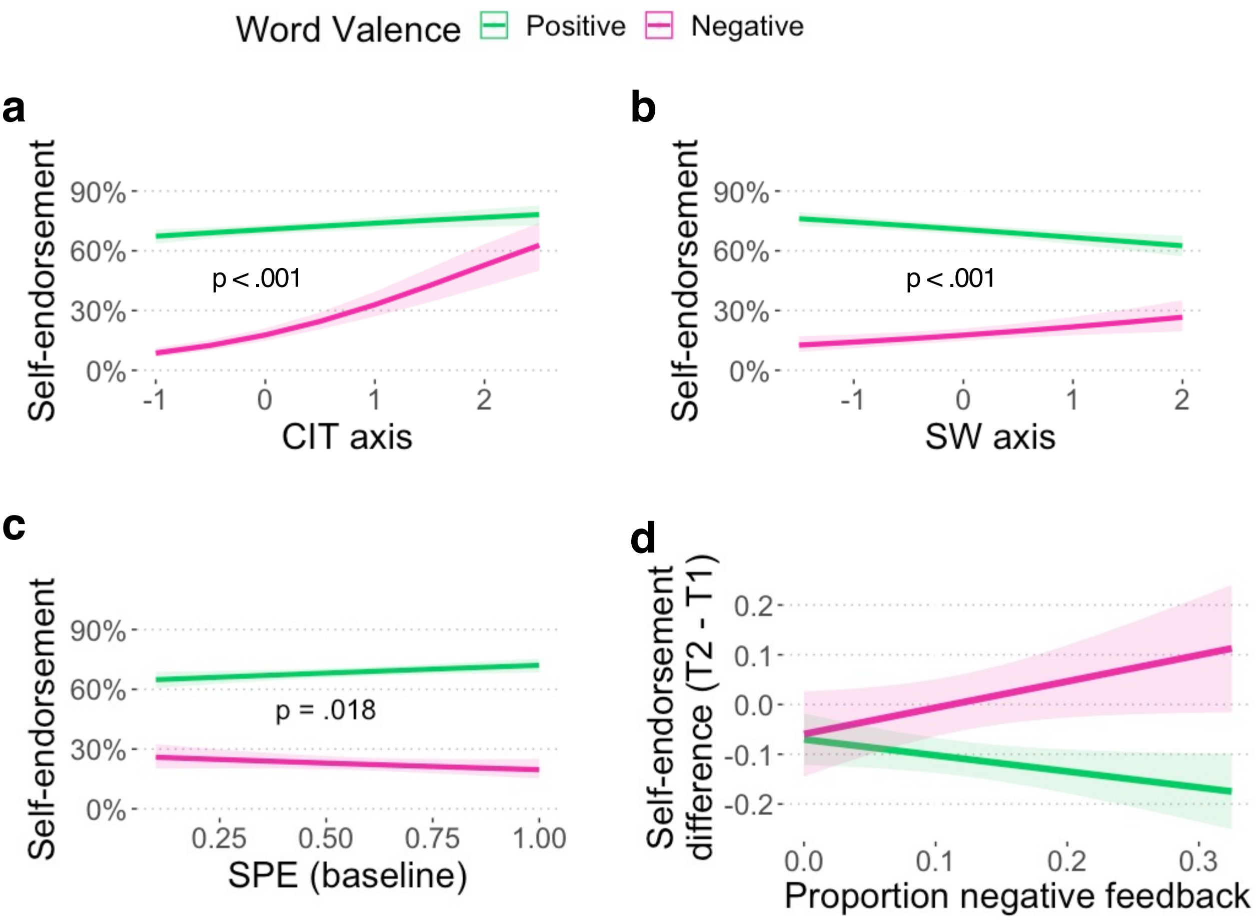

Supplementary Figure 14. Valence-specific relationship of self-endorsements for positive and negative word during the self-referential encoding task in Exp 2 (N = 335) plotted as marginal effects from a logistic regression showing that **a)** Compulsivity and Intrusive Thought transdiagnostic axis is positively related negative word endorsement, and **b)** Social Withdrawal transdiagnostic axis is both negatively related to positive word endorsement and positively related negative word endorsement. Similar plots in Exp 2 for **c)** global SPEs during baseline showing that they are associated positively with positive and negatively with negative word endorsements. **d)** Marginal effects of the interaction of proportion of negative feedback and word valence on the self-endorsement difference between SRET timepoint 2 – timepoint 1. The difference in self-endorsement increases for negative and decreases for positive words with the increase in the proportion of trials on which negative feedback was delivered on the previous task block. For all panels, shaded error bars show 95% bootstrapped confidence intervals, statistical comparisons were performed using mixed regressions, and p-values were obtained through likelihood ratio tests with χ^2 distributions.

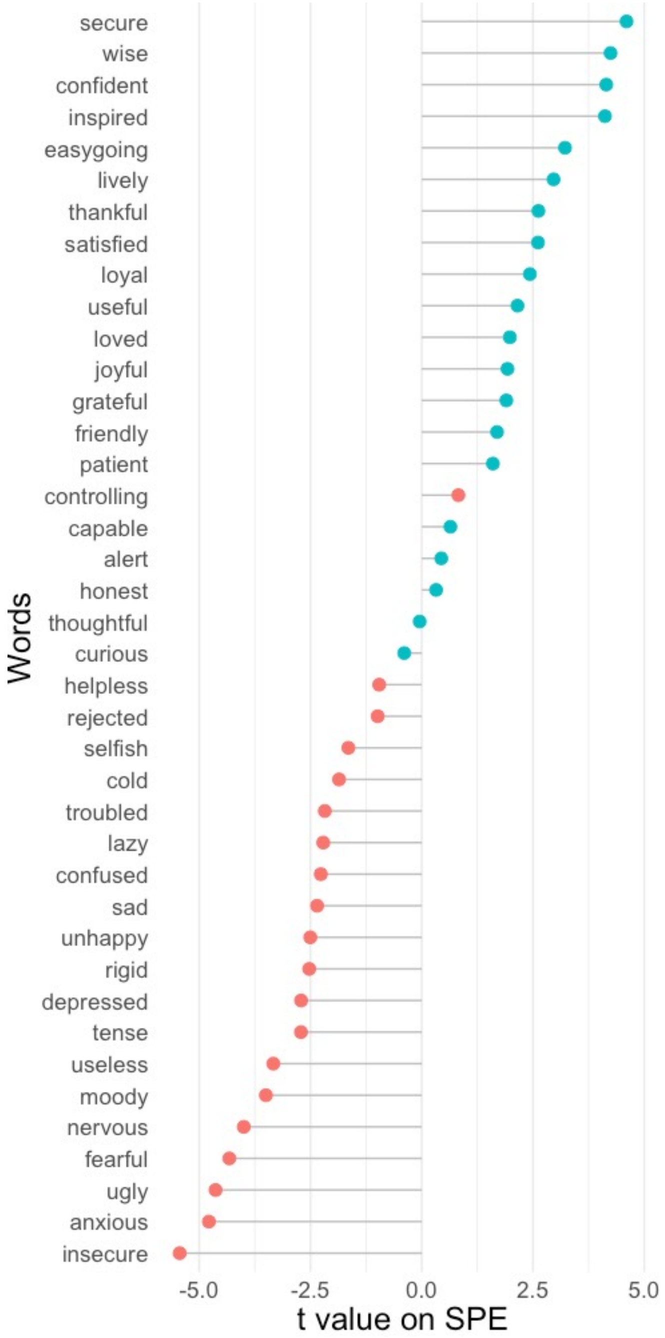

Supplementary Figure 15. t values of for the regression of each positive (blue) and negative (red) word endorsement upon SPE during baseline blocks of Exp 1

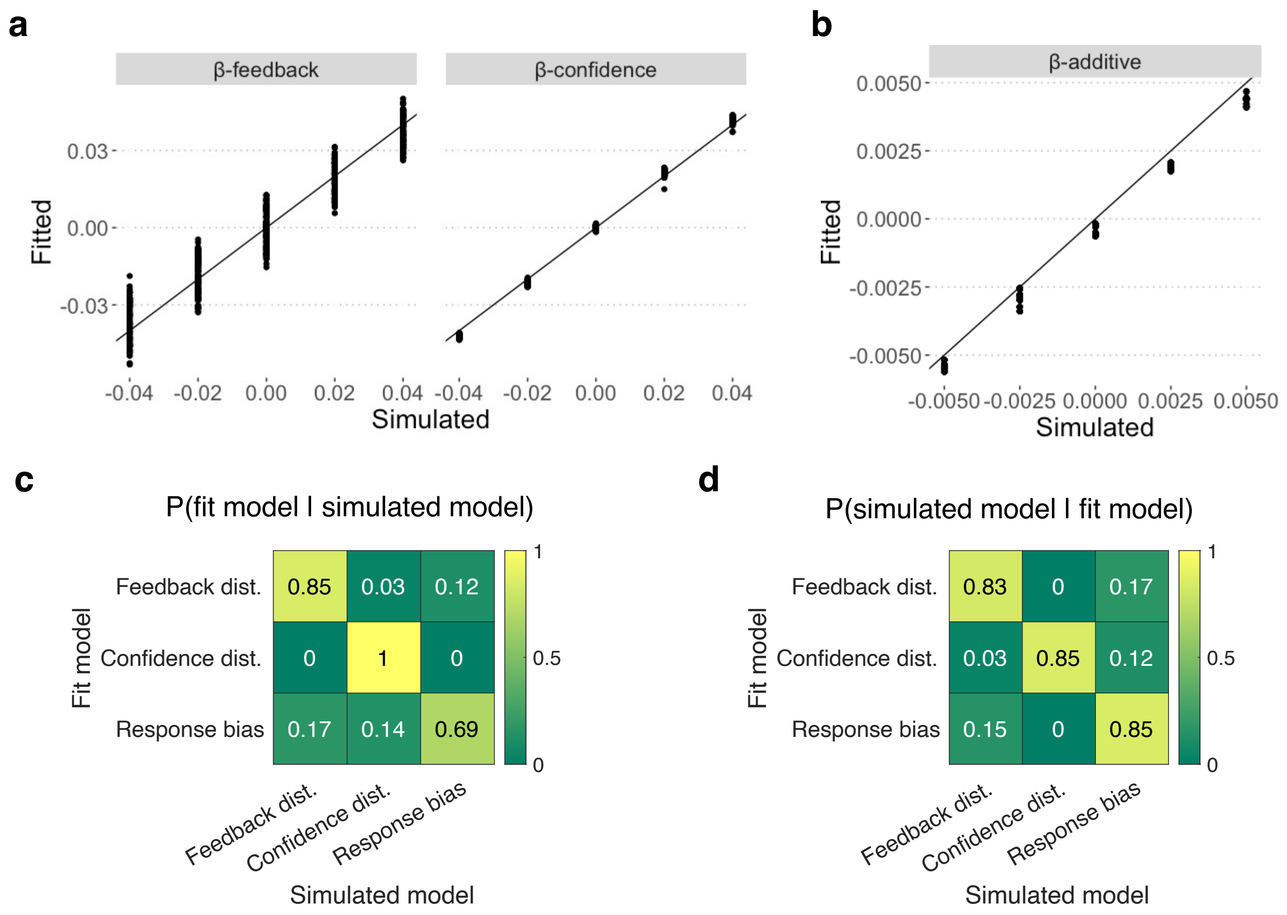

Supplementary Figure 16. Parameter recovery of regression slope parameters linking symptoms to **a)** feedback distortion (left) and confidence distortion (right), and **b)** response bias. Simulated values on x-axis and model recovered values on y-axis. **c)** Confusion matrix of model recovery depicting the probabilities of the best fitting distortion model based on deviation information criterion (DIC) given which of the three distortions was simulated. **d)** Inversion matrix of model recovery depicting the probabilities for which of the three distortions was simulated given a best-fitting distortion.

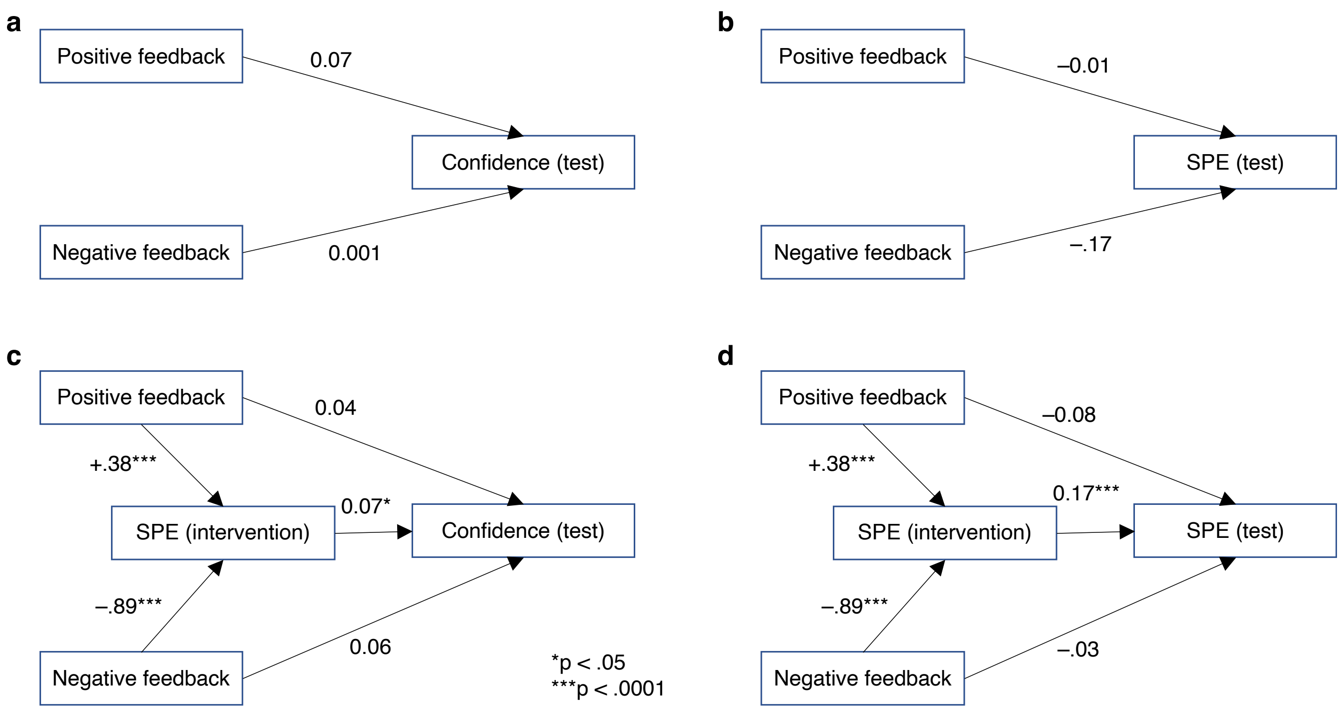

Supplementary Figure 17. A depiction of mediation analyses of positive and negative feedback's effect on test-block local confidence and test-block SPE by intervention-block SPE in Exp 1. Direct effect of feedback on test-block **a)** local confidence and **b)** SPE. Indirect effect of feedback on test-block **c)** local confidence and **d)** SPE, that both were fully mediated by SPE of the intervention block.

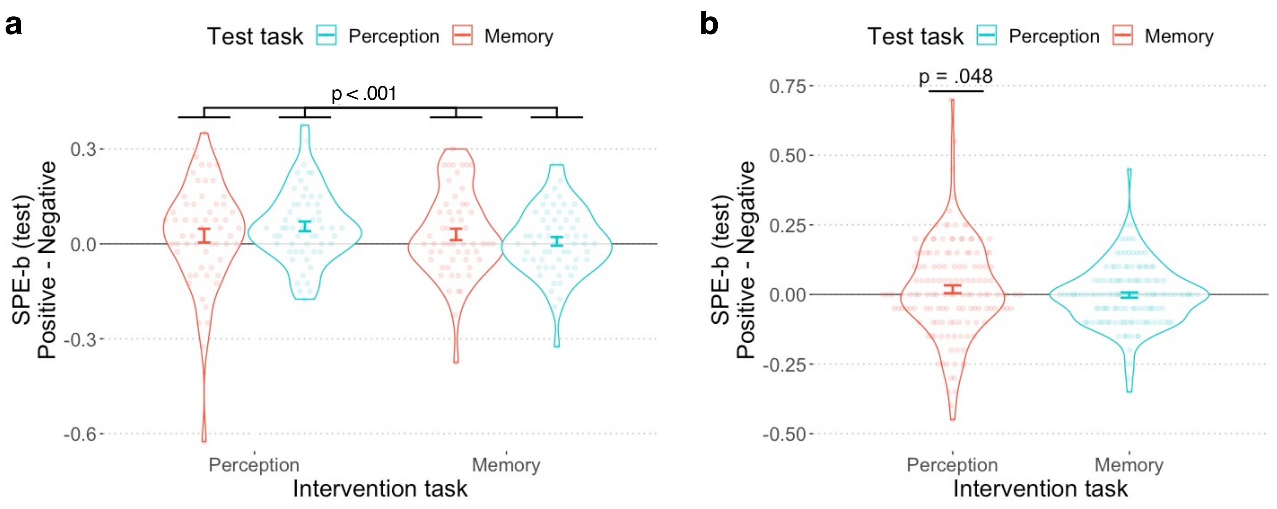

Supplementary Figure 18. Difference in SPEs between test blocks following positive and negative intervention blocks plotted separately for the two intervention tasks and two test tasks in **a)** Exp 1 (N = 230), and **b)** Exp 2 (N = 278). Error bars show 95% bootstrapped confidence intervals, statistical comparisons were performed using mixed regressions, and p-values were obtained through likelihood ratio tests with χ^2 distributions.

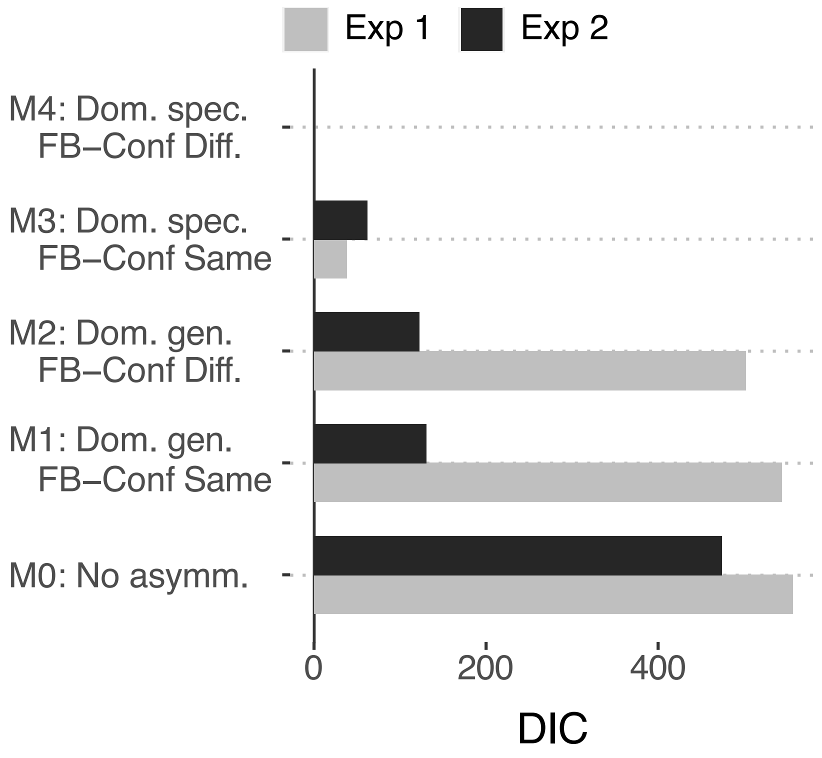

Supplementary Figure 19. DIC values for five models relating local confidence and performance feedback fit to the SPE data. In both Exp 1 and Exp 2, Model 4, which contained separate asymmetry parameters for feedback and confidence that were domain specific (i.e., separate for memory and perception) provided the best fit.

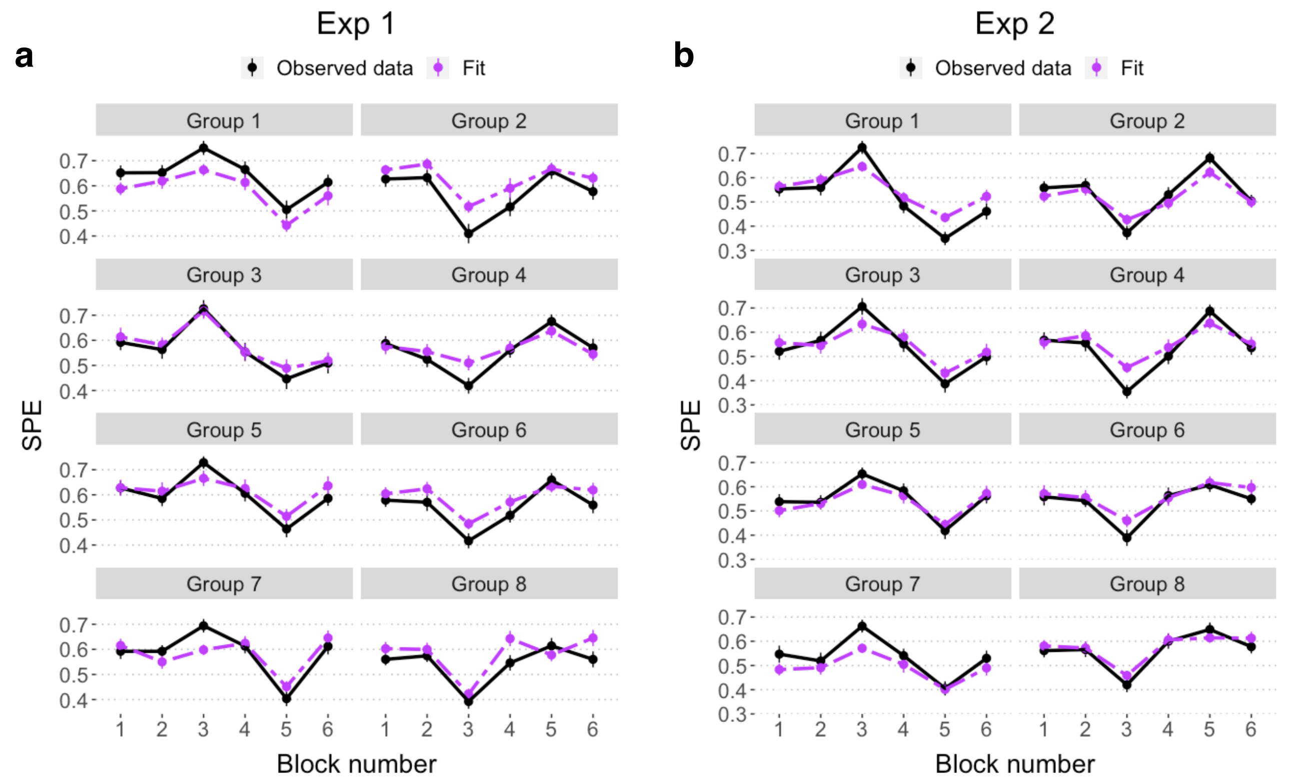

Supplementary Figure 20. SPE values (black) across the six task blocks averaged for participants belonging to groups that received positive feedback on block 3 and negative feedback on block 5 (i.e., Group 1, 3, 5, 7) shown in the top panel and those that received negative feedback on block 3 and positive feedback on 5 (Groups 2, 4, 6, 8) shown in the bottom panel for **a)** Exp 1 (N = 230), and **b)** Exp 2 (N = 278). Dashed purple lines are model fits. Error bars show SEMs.

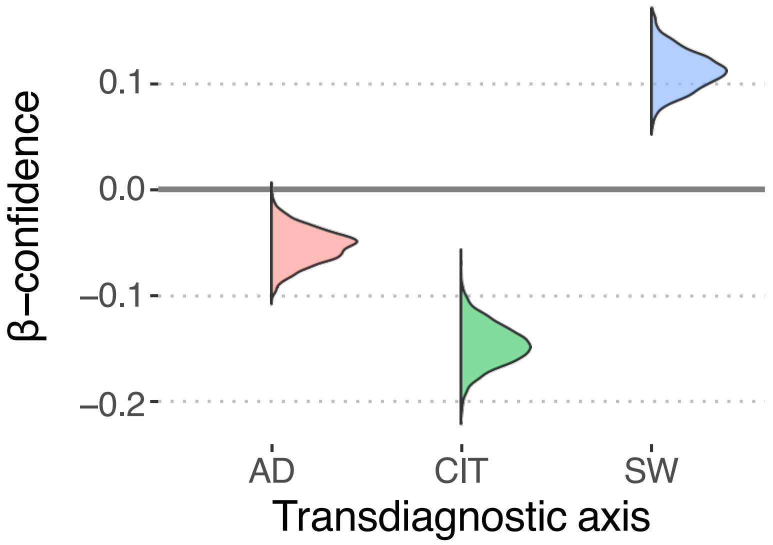

Supplementary Figure 21. Posterior distributions of the regression slope parameter for distortion in using local confidence to form global SPEs along the three transdiagnostic axes.

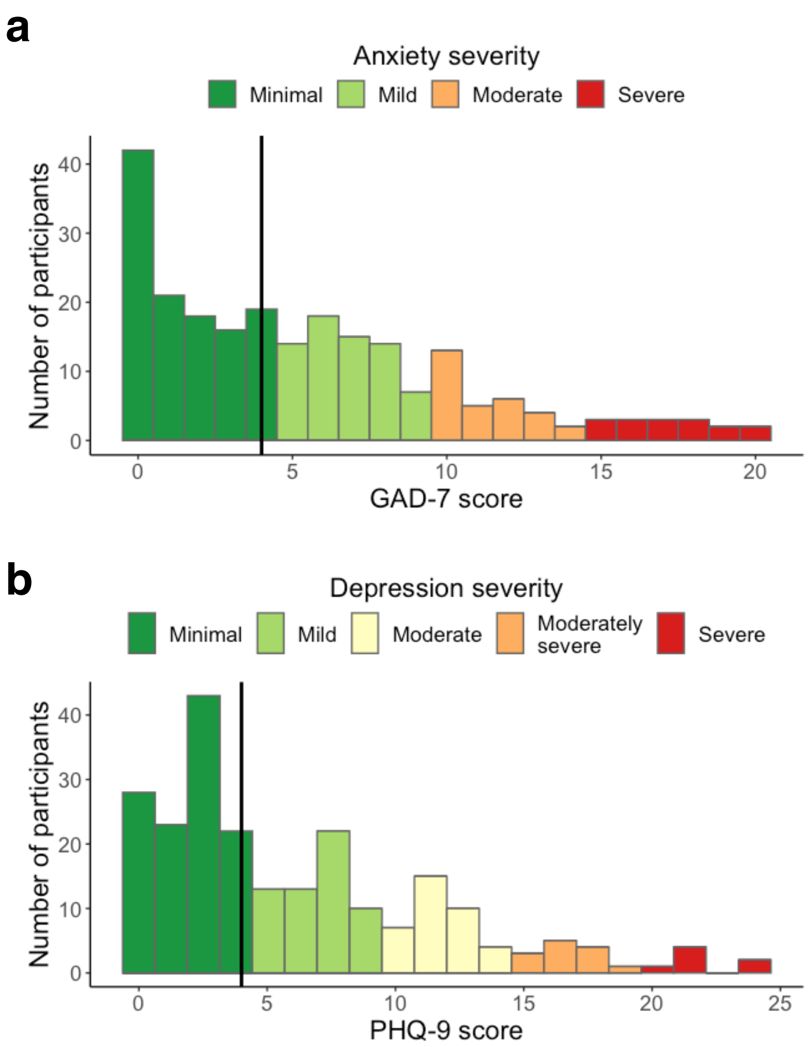

Supplementary Figure 22. Histograms of **a)** general anxiety scores measured by GAD-7 and **b)** depression scores measured by PHQ-9 in the Exp 1 sample (N = 230). Cutoffs for severity levels of anxiety and depression scores is indicated in each plot in different colours. Black vertical line shows the median score in the sample for each questionnaire.

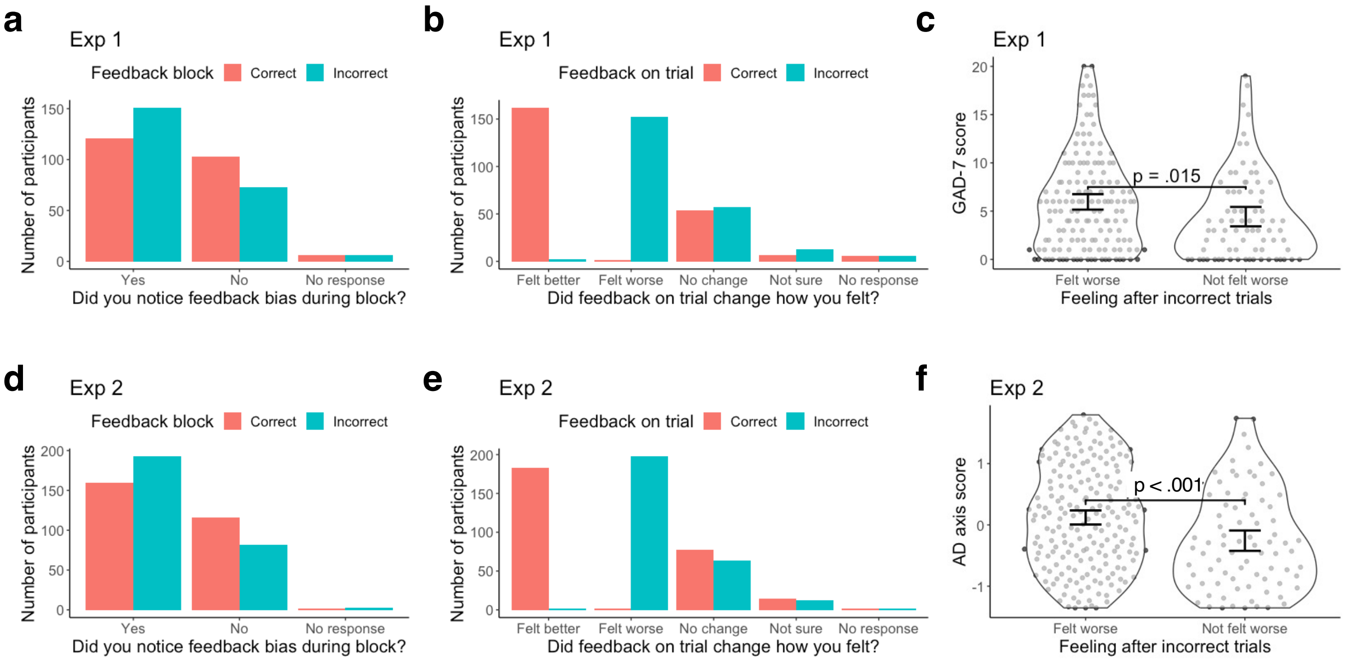

Supplementary Figure 23. **a, d)** The number of participants who reported noticing that one of the blocks had biased positive feedback (more feedback on correct trials) and negative feedback (more feedback on incorrect trials) in Exp 1 (N = 230) and Exp 2 (N = 278). **b, e)** Number of participants who reported different changes in how they felt after feedback on correct and incorrect trials in Exp 1 an Exp 2. **c, f)** Anxiety scores in Exp 1 and Anxious-Depression axis scores in Exp 2 of participants who reported feeling worse after incorrect trials compared to those who reported not feeling worse. Error bars show 95% bootstrapped confidence intervals, statistical comparisons were performed using mixed regressions, and p-values were obtained through likelihood ratio tests with χ^2 distributions.

Supplementary References

1. Kumle, L., Võ, M. L.-H. & Draschkow, D. Estimating power in (generalized) linear mixed models: An open introduction and tutorial in R. *Behav. Res. Methods* **53**, 2528–2543 (2021).
2. Seow, T. X. F., Rouault, M., Gillan, C. M. & Fleming, S. M. How local and global metacognition shape mental health. *Biol. Psychiatry* (2021) doi:10.1016/j.biopsych.2021.05.013.
3. Gillan, C. M., Kosinski, M., Whelan, R., Phelps, E. A. & Daw, N. D. Characterizing a psychiatric symptom dimension related to deficits in goal-directed control. *eLife* **5**, e11305 (2016).
4. Hopkins, A. K., Gillan, C., Roiser, J., Wise, T. & Sidarus, N. Optimising the measurement of anxious-depressive, compulsivity and intrusive thought and social withdrawal transdiagnostic symptom dimensions. Preprint at https://doi.org/10.31234/osf.io/q83sh (2022).
5. Rouault, M., Seow, T., Gillan, C. M. & Fleming, S. M. Psychiatric Symptom Dimensions Are Associated With Dissociable Shifts in Metacognition but Not Task Performance. *Biol. Psychiatry* **84**, 443–451 (2018).
6. Hoven, M., Luigjes, J., Denys, D., Rouault, M. & van Holst, R. J. How do confidence and self-beliefs relate in psychopathology: a transdiagnostic approach. *Nat. Ment. Health* **1**, 337–345 (2023).
7. Kroenke, K. & Spitzer, R. L. The PHQ-9: A New Depression Diagnostic and Severity Measure. *Psychiatr. Ann.* **32**, 509–515 (2002).

8. Lau, H. & Maniscalco, B. A signal detection theoretic approach for estimating metacognitive sensitivity from confidence ratings. *Conscious. Cogn.* **21**, 422–430 (2012).

9. Fleming, S. M. HMeta-d: hierarchical Bayesian estimation of metacognitive efficiency from confidence ratings. *Neurosci. Conscious.* **2017**, (2017).

10. Benwell, C. S. Y., Mohr, G., Wallberg, J., Kouadio, A. & Ince, R. A. A. Psychiatrically relevant signatures of domain-general decision-making and metacognition in the general population. *Npj Ment. Health Res.* **1**, 1–17 (2022).
