# Supplementary material for: Distorted learning from local metacognition supports transdiagnostic underconfidence
Source: Nat Commun. 2025 Feb 21;16:1854. doi: 10.1038/s41467-025-57040-0 (PMC11845503; doi:10.1038/s41467-025-57040-0)
Supplement: Supplementary file 3 — Reporting Summary [file 41467_2025_57040_MOESM3_ESM.pdf]

nature portfolio

Corresponding author(s): Sucharit Katyal

Last updated by author(s): Dec 4, 2024

# Reporting Summary

Nature Portfolio wishes to improve the reproducibility of the work that we publish. This form provides structure for consistency and transparency in reporting. For further information on Nature Portfolio policies, see our Editorial Policies and the Editorial Policy Checklist.

## Statistics

For all statistical analyses, confirm that the following items are present in the figure legend, table legend, main text, or Methods section.

| n/a | Confirmed | |
|---|---|---|
| ☐ | ☒ | The exact sample size (*n*) for each experimental group/condition, given as a discrete number and unit of measurement |
| ☐ | ☒ | A statement on whether measurements were taken from distinct samples or whether the same sample was measured repeatedly |
| ☐ | ☒ | The statistical test(s) used AND whether they are one- or two-sided<br>*Only common tests should be described solely by name; describe more complex techniques in the Methods section.* |
| ☐ | ☒ | A description of all covariates tested |
| ☐ | ☒ | A description of any assumptions or corrections, such as tests of normality and adjustment for multiple comparisons |
| ☐ | ☒ | A full description of the statistical parameters including central tendency (e.g. means) or other basic estimates (e.g. regression coefficient) AND variation (e.g. standard deviation) or associated estimates of uncertainty (e.g. confidence intervals) |
| ☐ | ☒ | For null hypothesis testing, the test statistic (e.g. *F*, *t*, *r*) with confidence intervals, effect sizes, degrees of freedom and *P* value noted<br>*Give P values as exact values whenever suitable.* |
| ☐ | ☒ | For Bayesian analysis, information on the choice of priors and Markov chain Monte Carlo settings |
| ☐ | ☒ | For hierarchical and complex designs, identification of the appropriate level for tests and full reporting of outcomes |
| ☐ | ☒ | Estimates of effect sizes (e.g. Cohen's *d*, Pearson's *r*), indicating how they were calculated |

*Our web collection on statistics for biologists contains articles on many of the points above.*

## Software and code

Policy information about availability of computer code

**Data collection**

Study was programmed in Javascript using the Phaser 3 toolbox in VS Code v1.75.0. Stimuli for the study available at Github https://github.com/sucharitk/confidence-distortion-AD/

**Data analysis**

Bayesian model implemented using the JAGS 3.4.0 toolbox in MATLAB 2022b;
All other analysis was performed in (incuding generation of manuscript figures) RStudio 2022.07.2
For analysis in R, we used the following packages:
lmerTest package (version 3.1-3)
stats package (version 4.1.0)
mediation package (version 4.5.0)
sjPlot package (version 2.8.11)
effectsize (version 0.8.3)
BayesFactor package (version 0.9.12-4.7)
emmeans package (version 1.10.1)

For manuscripts utilizing custom algorithms or software that are central to the research but not yet described in published literature, software must be made available to editors and reviewers. We strongly encourage code deposition in a community repository (e.g. GitHub). See the Nature Portfolio guidelines for submitting code & software for further information.

# Data

Policy information about availability of data

All manuscripts must include a data availability statement. This statement should provide the following information, where applicable:

- Accession codes, unique identifiers, or web links for publicly available datasets
- A description of any restrictions on data availability
- For clinical datasets or third party data, please ensure that the statement adheres to our policy

Data for this study is publicly available at https://github.com/sucharitk/confidence-distortion-AD/

Data availability
Aggregated data for reproducing all results and figures are publicly available at https://github.com/sucharitk/confidence-distortion-AD/

# Research involving human participants, their data, or biological material

Policy information about studies with human participants or human data. See also policy information about sex, gender (identity/presentation), and sexual orientation and race, ethnicity and racism.

| | |
|---|---|
| Reporting on sex and gender | We asked participants to report their self-identified gender that included an option for choosing nonbinary. Gender data is available in the source data and gender counts for the two samples are provided in the Methods section.<br>Gender-segregated analyses were not performed as they were not part of our hypotheses. Any posthoc analyses segregated by gender would not be meaningful due to insufficient sample size.<br>Following previous work on the relationship between confidence and transdiagnostic symptoms we controlled for gender in our regression analyses. |
| Reporting on race, ethnicity, or other socially relevant groupings | N/A |
| Population characteristics | In the first dataset (Exp 1), we measured depression, general anxiety and social anxiety scores for each participant using standardised questionnaires (PHQ-9, GAD-7, and mini-SPIN). Data was analysed as a linear regression within our Bayesian model using these scores.<br><br>In the second dataset (Exp 2), we measured symptom scores transdiagnostically using a 71-questionnaire battery and factor separation method developed by Hopkins et al, (2022); https://psyarxiv.com/q83sh/. Through this procedure, we obtained scores for each participant along 3 transdiagnostic axes: Anxious-Depression, Compulsivity & Intrusive Thought, and Social Withdrawal. These scores were entered as regressors in our Bayesian model. |
| Recruitment | Participants were recruited via the online platform Prolific. This limits our sample to those having internet access and possessing internet literacy. |
| Ethics oversight | Study approved by the UCL Research Ethics Committee; approval number 21029/001 |

Note that full information on the approval of the study protocol must also be provided in the manuscript.

# Field-specific reporting

Please select the one below that is the best fit for your research. If you are not sure, read the appropriate sections before making your selection.

☐ Life sciences ☒ Behavioural & social sciences ☐ Ecological, evolutionary & environmental sciences

For a reference copy of the document with all sections, see nature.com/documents/nr-reporting-summary-flat.pdf

# Behavioural & social sciences study design

All studies must disclose on these points even when the disclosure is negative.

| | |
|---|---|
| Study description | The study tests how the formation of global confidence in performing gamified computer tasks is distorted in individuals with anxious-depression symptoms. We hypothesised that global confidence may be distorted due to greater learning from negative vs. positive feedback and/or greater learning from low vs. high local confidence trials. This is an experimental quantitative study. |
| Research sample | Participants were recruited online using the Prolific (prolific.com) platform. After dropouts and exclusions, a total of 230 participants remained for Exp 1 (age mean and SD = 32 ± 9; gender: 127 females, 185 males, 2 nonbinary) and 278 participants for Exp 2 (age mean and SD = 32 ± 9; gender: 176 females, 278 males, 3 nonbinary). |
| Sampling strategy | The research sample was a “convenience sample” recruited from the Prolific online platform. We chose this method to allow |

| | |
|---|---|
| Sampling strategy | collection of a large dataset for evaluating relationships between task measures and subclinical symptoms.<br>For the first dataset (Exp 1), we used an exploratory sample size of a minimum of 25 participants per 8 randomised groups, which with the randomisation procedure and exclusions resulted in N = 230.<br>For the second dataset (Exp 2), we estimated the sample size to ensure power > 0.9 for each preregistered hypotheses. Effect sizes for estimating power were determined based on Exp 1. |
| Data collection | Data was collected online on participants' personal computers and saved on an online database. Due to the remote nature of the testing procedure we cannot determine if another individual besides the participant was present during data collection. The researcher was not present at the location of data collection and was blind to group allocation. |
| Timing | Exp 1 – start: 5 Aug 2022 (12:30), end: 18 Aug 2022<br>Exp 2 – start 20 Jan 2023 (16:37), end: 27 Jan 2023 |
| Data exclusions | Participants were excluded from all analyses if they missed one of the three “catch” questions. Two catch questions were administered during the self-referential encoding task (SRET). Here participants were shown the words ‘human and ‘keyboard’ randomly placed between the other 20 positive and negative adjectives; participants were excluded if they did not self-endorse these words maximally (‘Yes’ in Exp 1 and >.875 on the slider in Exp 2) and minimally (‘No’ in Exp 1 and <.125 on the slider in Exp 2) respectively. Additionally, embedded within mental health questionnaires, participants were asked “I take astronaut missions to space” and were excluded if they did not select the option “Never.” In all, 14 participants were excluded in Exp 1 and 71 participants in Exp 2 for missing at least one catch question.<br><br>For analyses involving local confidence and global self-performance estimates, we also excluded participants, 1) whose performance was outside the interval (.60 .85] on any one of the 6 task blocks (Exp 1: 32 participants, Exp 2: 99 participants), 2) who did not exhibit sufficient variability in trial-by-trial confidence ratings defined as having <.05 SD across trials for each task (on a continuous confidence scale of 0—1; Exp 1: 9 participants, Exp 2: 12 participants), and 3) who did not have stable behavioural staircases in the perception/memory tasks as assessed visually (Exp 1: 19 participants, Exp 2: 0 participants). Finally, one participant from Exp 2 was excluded because their questionnaire data did not get saved on our database (possibly due to an internet issue at their end).<br><br>Note that the sample size in Exp 1 deviated slightly from our preregistered analysis. In the preregistered analysis we excluded participants whose performance was outside the closed interval (.60 .85), whereas for the reported analyses here we used a part open interval at the upper end (i.e., we included participants whose performance was exactly .85 on a block). This decision was made prior to data analysis for Exp 2 and was done because unlike Exp 1 where all blocks had 40 trials (and each correct trial would correspond to a .025 increment in accuracy), in Exp 2 the test blocks had only 20 trials (each correct trial corresponding to a .05 increase in accuracy). This would result in a more stringent exclusion criterion in Exp 2 as participants would be excluded from the study for getting only 3 (vs. 6) wrong responses on even one of the two test blocks. Consistent with this idea, we found that the preregistered criterion resulted in a performance-based exclusion of 39% of the participants compared to our expected performance-based exclusion rate of 18% from Exp 1. However, we find that even if we use the more stringent (i.e., preregistered) exclusion criterion, all our key results – namely feedback manipulation of SPE, greater sensitivity of SPEs to low vs. high confidence with higher anxious-depression scores, lack of difference in sensitivity of SPEs to negative vs. positive feedback with higher anxious-depression scores – remain the same in both Exp 1 and 2.<br><br>In Exp 1, 19 participants were excluded as their behavioural staircases had not stabilised (no such participants were excluded in Exp 2). This was likely due to a minor error in the stimulus code, as follows. For the practice block we used a staircase with two starting “jumps” where the staircase was incremented by two steps instead of one if participants were incorrect on the first two trials. We did not intend to use these jumps for the main task blocks. However, for the first half of the participants in Exp 1 these jumps were erroneously kept as part of the main task code. This appeared to impact staircase stabilisation in a few participants who got both the initial trials incorrect who were thus excluded from analyses. For the second half of participants in Exp 1 and for all participants in Exp 2 this error was rectified.<br><br>The three performance-/confidence-based exclusion criteria used in the confidence analysis above were not relevant (and overly stringent) for analyses involving the self-referential encoding task (SRET). Instead, for the SRET, we used a less stringent criterion of excluding participants whose mean accuracy was below .6 across all blocks on average (and were thus presumably not paying sufficient attention to the experiment). Additionally, for this task we found that some subjects had extremely long RTs ranging from 10 seconds to several minutes. Such participants were presumably also not doing the task sincerely and would especially add noise to the pre-post feedback intervention changes in self-beliefs we aimed to test in Exp 2. We thus removed participants for whom any word RT was greater than 5 IQRs from the median (~4.9 sec). The results were not substantially impacted by the specific criterion – similar results were obtained, for example, if we used stricter (e.g., >3 IQRs or ~3.2 sec) or more relaxed (e.g., >7 IQRs or ~6.4 sec) exclusion criteria. Final sample sizes for SRET were N = 300 for Exp 1 and N = 335 for Exp 2. |
| Non-participation | 76 participants dropped out in Exp 1 and 131 participants dropped out in Exp 2. We did not collect participants' reasons for dropping out. |
| Randomization | Randomisation in both experiments was performed by allocating each subsequent participant to one of eight groups using a pre-assigning randomised sequence. Additional participants beyond the length of the pre-assigned sequence were 1) in Exp 1 randomly assigned to those groups that had not reached our preset criterion of 25 participants per group, until this criterion was reached, and 2) in Exp 2 randomly assigned to one of the eight groups.<br><br>Further details of the randomisation procedure are provided in the Supplementary Material Methods section. |

# Reporting for specific materials, systems and methods

We require information from authors about some types of materials, experimental systems and methods used in many studies. Here, indicate whether each material, system or method listed is relevant to your study. If you are not sure if a list item applies to your research, read the appropriate section before selecting a response.

## Materials & experimental systems

| n/a | Involved in the study |
|---|---|
| ☒ | ☐ Antibodies |
| ☒ | ☐ Eukaryotic cell lines |
| ☒ | ☐ Palaeontology and archaeology |
| ☒ | ☐ Animals and other organisms |
| ☒ | ☐ Clinical data |
| ☒ | ☐ Dual use research of concern |
| ☒ | ☐ Plants |

## Methods

| n/a | Involved in the study |
|---|---|
| ☒ | ☐ ChIP-seq |
| ☒ | ☐ Flow cytometry |
| ☒ | ☐ MRI-based neuroimaging |
